# Bioactive Extracts from Yucatán Marine Invertebrates as Modulators of Microalgal Dynamics

**DOI:** 10.1007/s10126-026-10630-5

**Published:** 2026-05-19

**Authors:** Dawrin Pech-Puch, Rita Pires, Bárbara A. Rebelo, Diana Grilo, Carlos Gonzáles-Salas, Susana Eunice Calva-Pérez, Susana M. Gomes, Harold Villegas-Hernández, Sergio Guillén-Hernández, Abel M. Forero, Jaime Rodriguez, Carlos Jiménez, Oscar A. Lenis-Rojas, Rita Abranches

**Affiliations:** 1 Escuela Nacional de Estudios Superiores Unidad Mérida (ENES Mérida), Universidad Nacional Autónoma de México (UNAM), Carretera Mérida-Tetiz km 4.5, Tablaje, Catastral No. 6998, Municipio de Ucú, Ucú, CP 97357 Merida Mexico; 2 Instituto de Tecnologia Química e Biológica António Xavier (ITQB NOVA), Universidade Nova de Lisboa, Lisbon, Portugal; 3 Departamento de Biología Marina, Universidad Autónoma de Yucatán, Km. 15.5, Carretera Mérida-Xmatkuil, A.P. 4-116 Itzimná, Merida, CP 97100 Mexico; 4 Centro de Interdisciplinar de Química e Bioloxía (CICA), Facultade de Ciencias, Universidade da Coruña, 15071 A Coruña, Spain

**Keywords:** Sponges, Bioactive compounds, Arenosclerin A and C, *Haliclona* (*Rhizoniera*) *curacaoensis*, *Halichondria melanadocia*, Algicidal, Stimulants

## Abstract

The marine environment is a rich source of bioactive compounds with a wide range of applications. In this study, 65 extracts from sponges, ascidians, and a gorgonian collected from the Yucatán Peninsula, Mexico, were evaluated for their modulatory effects on the growth of four microalgal species. Some extracts were found to exhibit pronounced algicidal activity, while others promoted growth. Bioassay-guided fractionation of the extract from *Haliclona (Rhizoniera) curacaoensis* led to the identification of arenosclerins A and C as potent algicidal agents, active across all tested microalgae. In contrast, the extract from *Halichondria melanadocia* enhanced microalgal growth, and chemical analysis revealed the presence of medelamine A and B as putative growth-promoting compounds. This study provides a new report on microalgae-modulating activities of marine sponge metabolites. The dual activity observed, both inhibitory and stimulatory, not only contributes to our understanding of marine compound diversity but also underscores the potential of these marine-derived compounds for sustainable environmental management practices.

**Supplementary Information:**

The online version contains supplementary material available at 10.1007/s10126-026-10630-5.

## Introduction

Climate change has triggered transformations in coastal ecosystems, leading to warming, acidification, and deoxygenation of aquatic ecosystems (Gobler 2020). These changes have caused diverse environmental problems, including loss of seagrass habitats, coral reef degradation, marine mammal deaths, shellfish poisonings, and the increasing number and intensity of harmful algal blooms (HABs) (Masó and Garcés 2006; Zingone and Oksfeldt Enevoldsen 2000).

Artificial algicides (CuSO_4_, Cu^2+^ complexes, dibromohydantoin, diuron, and surfactants) are effective in rapidly eliminating harmful algal blooms, and their use may adversely affect non-target aquatic organisms and ecosystem functioning (Ghoran et al. 2022). A promising approach to mitigate or prevent HABs is the application of naturally occurring species or their bioactive compounds (Simons et al. 2021). Compounds from fungi, such as those isolated from *Aspergillus* sp. and marine sponge-associated fungi, like anthraquinones have demonstrated algicidal properties (Ghoran et al. 2022, 2023). Similarly, methanol and water-soluble extracts from various seaweeds from Korean coasts have been reported to display algicidal effects on various red tide microalgae (Jeong et al. 2000). The algicidal effect of the marine bacteria *Kordia algicidal* against various alga species and *Qipengyuania* sp. 3-20A1M, isolated from surface seawater, against harmful *Margalefidinium polykrikoides* were reported recently (Jeong et al. 2000). The marine environment harbors an estimated 80% of the world’s plant and animal species and has been a prolific source of active metabolites (Mayer et al. 2024). Particularly, sponges and tunicates are sessile organisms that have evolved and developed chemical defense mechanisms, leading to the generation of a diverse array of specialized metabolites that can also act as signal molecules with chemoecological roles (Chaves-Fonnegra et al. 2008; Mehbub et al. 2014).

The metabolites produced by sponges that are not directly involved in normal growth can be classified into several categories, including alkaloids, terpenoids, glycosides, phenols, phenazines, polyketides, fatty acid products, peptides, amino acid analogues, nucleosides, porphyrins, and aliphatic cyclic peroxides (Mehbub et al. 2014; Thakur and Singh 2016). Alkaloids have a wide range of chemical structures and exist in derivatives of several heterocyclic rings. Increasing evidence indicates a wide distribution of piperidine and pyridine alkaloids in marine sponges of five genera, including *Amphimedon*, *Cribrochalina*, *Haliclona*, *Niphates* and *Xestospongia* (Zhang et al. 2016). Extracts and isolated compounds from *Haliclona* sp. have been tested for cytotoxicity against cancer cell lines or antimicrobial activity (Bian et al. 2020; Pech-Puch et al., 2020a; Zhang et al. 2016). Examples of alkaloids which have been tested for their antimicrobial activity and cytotoxicity are arenosclerins A and C, tetracyclic alkylpiperidines isolated from the marine sponge *Arenosclera brasiliensis* (Torres et al. 2000, 2002). In these studies, the broad activities presented by *A*. *brasiliensis* alkaloids suggested a defensive role of arenosclerins against microbial infection and potential predators. Sponge organisms appear to defend themselves against infections by producing or accumulating secondary metabolites. These alkaloids present a broad cytotoxic activity, rather than a particular mode of action because they were active towards cancer cells as well as normal cells, in equal concentration ranges (Torres et al. 2002).

Although marine alkaloid compounds have been tested against various microorganisms, their impact on the marine environment remains unexplored. Microalgal models have been used to test the microalgal growth-inhibition activities of marine natural products, due to their sensitivity (greater than invertebrates and fish) to various pollutants, ease of laboratory cultivation, rapid growth rate, and short life cycle (Castro et al. 2021; Zhao et al. 2018). Such model species are commonly used as preliminary screening systems to evaluate algal growth-inhibitory effects relevant to the development of metabolite-based strategies for HAB control (Zhu et al. 2021).

On the other hand, compounds that promote algae growth hold promise for sustainable agriculture practices. Algae have a remarkable ability to absorb nutrients and contaminants from their surroundings, making them potential agents for cleaning polluted water bodies (Alazaiza et al. 2022; Hennequin et al. 2022) and an alternative in bioremediation processes (Balzano et al. 2020). Additionally, algae-based fertilizers can enhance soil fertility and crop productivity while reducing the reliance on synthetic fertilizers, which can contribute to water pollution and soil degradation (Alazaiza et al. 2022; Alvarenga et al. 2023; Hawrot-Paw et al. 2019). One of the primary reasons for the importance of these compounds lies in their role in bioremediation. Beyond their potential environmental and economic benefits and applications, these compounds are crucial for the development of renewable energy sources. Algae are highly efficient in converting sunlight and carbon dioxide into biomass through photosynthesis, making them a promising source of biofuels, such as biodiesel and bioethanol (Alazaiza et al. 2022; Yang et al. 2011). Furthermore, algae are rich sources of protein, vitamins, minerals, and essential fatty acids (Wu et al. 2023), making them valuable ingredients for functional foods, dietary supplements, and animal feed (Mendes et al. 2022). By supporting algae growth, such compounds may offer environmental, economic, and nutritional benefits across various sectors.

In this study, 65 marine extracts from 51 sponges, 13 ascidians, and 1 gorgonian were tested for their impact on the growth of four microalgae species, *Phaeodactylum tricornutum*, *Isochrysis galbana*, *Haematococcus pluvialis*, and *Tetraselmis chuii*. The objective was to identify extracts with growth-inhibiting or growth-promoting activity. Two active extracts were selected for further investigation to find the potential compounds that enhance or inhibit the growth of algae. These findings highlight the potential of marine natural products, such as natural algicides for controlling HABs or microalgae growth promoters for applications in bioremediation, sustainable agriculture, renewable energy production, and food and nutrition sectors (Gaudêncio et al. 2023). By harnessing the natural capabilities of algae, marine natural compounds offer innovative solutions to pressing global challenges, towards a more sustainable and resilient future.

## Materials and Methods

### Animal Collection and Identification

Samples of animals were collected by snorkeling and SCUBA diving in different coastal zones of the Yucatán Peninsula, Mexico, during three different periods: September – December 2016, January – March 2017, and September 2018. The selected species were obtained from the following two regions: Mexican Caribbean (Cozumel Island, Rio Indio, Mahahual, and Bermejo, Quintana Roo) and Campeche Bank (Alacranes Reef and Progreso, Yucatán) (Pech-Puch et al., 2020a). A total of 65 organisms were collected and identified (Table S.1) using the following protocol:

i) Sponges. The organisms, once collected, underwent a fixation process in which each one of them was placed in vials containing 96% ethanol for 72 h. After fixation, one specimen from each organism was set aside for preservation and subsequent taxonomic identification, using a 4% formalin (CH_2_O) solution in seawater neutralized with sodium tetraborate (Na_2_[B_4_O_5_(OH)_4_]8H_2_O). The taxonomic identification of the collected species was carried out by biologist Patricia Gómez López from the Institute of Marine Sciences and Limnology at National Autonomous University of Mexico (ICMyL-UNAM), following the identification guide by Hooper and Van Soest (2002). Subsequently, the specimens were labeled, after a sample of each species was deposited in the Gerardo Green National Collection of the Phylum Porifera, housed at the ICMyL-UNAM in Mexico City.
ii) Ascidians. After the collection of the organisms, an individual from each species was anesthetized using menthol crystals dissolved in 96% ethanol and subsequently fixed in 4% formaldehyde (CH_2_O), following the protocol described by Monniot (1972). The dissection, staining with Harris hematoxylin, and taxonomic identification were performed by Dr. Elsa Vázquez from the University of Vigo, Spain. The process was carried out following the standard method and the tabular keys developed by Rocha et al. (2012).
iii) Gorgonians. The collected fragments were fixed in a 4% formaldehyde (CH_2_O) solution prepared with filtered seawater and neutralized with sodium tetraborate (Na_2_[B_4_O_5_(OH)_4_]8 H_2_O) to preserve the structure and composition of the tissue. Subsequently, the samples were transferred to containers with 70% ethanol (C_2_H_6_O) for long-term preservation. Species identification was carried out at the marine biology laboratory of the Autonomous University of Yucatán by Dr. Harold Villegas Hernández, using the taxonomic keys and identification guides specific to gorgonians of the Western Atlantic and Caribbean, as described by Bayer (1961) and Cairns (1977).

### Preparation of the Marine Organic Extracts

For the screening assays, tissue slices of the 65 species were extracted three times over 24-hour intervals, with a 500 mL mixture of dichloromethane–methanol (CH_2_Cl_2_-CH_3_OH (1:1 V/V)), at 25 °C. The solvent was then filtered and evaporated under vacuum at 40 °C using a rotatory evaporator. The resulting organic extracts were stored at − -20 °C in tightly sealed glass vials.

### Isolation of Arenosclerin A and C from *Haliclona* (*Rhizoniera*) *curacaoensis*.

Sliced bodies of *Haliclona* (*Rhizoniera*) *curacaoensis* sponge (wet weight, 595.0 g; dry weight, 371.5 g) were exhaustively extracted (3 times × 1.5 L) with CH_2_Cl_2_-CH_3_OH (1:1 V/V) mixture at 25 °C, as described above. The combined extracts were concentrated under reduced pressure to produce 7.9 g of a crude residue which was partitioned between CH_2_Cl_2_/H_2_O (1:1 V/V) to afford aqueous and organic phases following the standard fractionation methodology (Anta et al. 2002). The aqueous phase was extracted with *n*-butanol (200 mL) to yield 0.6 g of the final aqueous fraction (WW) and 0.4 g of the *n*-butanol fraction (WB), after removal of the solvents under reduced pressure. The organic phase was concentrated under reduced pressure and partitioned between 10% aqueous CH_3_OH (400 mL) and *n*-hexane (2 × 400 mL). The *n*-hexane fraction (FH) yielded 3.7 g, after removing the solvent under reduced pressure. The H_2_O content (% V/V) of the methanolic fraction was adjusted to 50% aqueous CH_3_OH, and the mixture was extracted with CH_2_Cl_2_ (100 mL). After removing the corresponding solvent under reduced pressure, 2.1 g and 1.1 g were obtained from the CH_2_Cl_2_ fraction (FD) and from the remaining aqueous methanolic fraction (FM), respectively. The extraction process yielded 5 fractions, as outlined in Table S.2.

The aqueous methanolic fraction (FM, 1.1 g) was submitted to Solid Phase Extraction (SPE) with a RP-18 cartridge (LC-18, 500 mg/3 x mL, Merck KGaA), using a discontinuous gradient from H_2_O to CH_3_OH and then CH_2_Cl_2,_ to give six subfractions FM (1–6). Fraction FM-2 eluted with H_2_O/CH_3_OH (2:1 V/V, 218.8 mg) was separated by Reversed-phase high-performance liquid chromatography (RP-HPLC). The mobile phase consisted of a 35 min gradient, transitioning from 10% to 25% of acetonitrile (MeCN) in H_2_O (V/V, each containing 0.04% trifluoroacetic acid). This was followed by an 18 min isocratic phase at 25% of MeCN, then a linear gradient from 25% to 100% of MeCN, and 5 min isocratic at 100% of MeCN at a flow rate of 2.0 mL/min. This process afforded the HPLC fraction FM-2-H2 at a retention time (Rt) of 9.5 min which gave 39.4 mg of arenosclerin A (**1**) after removing the solvent.

The CH_2_Cl_2_ fraction (2.1 g) was subjected to a SPE with RP-18 (Merck KGaA) using a discontinuous gradient from H_2_O to CH_3_OH and then CH_2_Cl_2_ to afford six subfractions FD (1–6). Fraction FD-3, eluted with H_2_O/CH_3_OH (1:1 V/V, 145.2 mg) was separated by RP-HPLC. The process involved a mobile phase consisting of 40 min linear gradient, from 25% to 50% of MeCN in H_2_O (V/V, each containing 0.04% trifluoroacetic acid). This was followed by a 15 min isocratic phase at 50% of MeCN, then a linear gradient from 50% to 100% of MeCN, and a 5 min isocratic at 100% of MeCN at a flow rate of 1.5 mL/min. This HPLC separation yielded the fraction FD-3-H1 at a retention time of 16.7 min, which afforded 50.6 mg of arenosclerin C (**2**) after removing the solvent.

The compounds were characterized by ^1^H, ^13^C, and 2D Nuclear magnetic resonance spectroscopy, and by high resolution electrospray mass spectrometry (HRESIMS).

### Dereplication of *Halichondria melanadocia* Organic Extract

Sliced bodies of the sponge *Halichondria melanadocia* (wet weight, 383.7 g; dry weight, 215.1 g) were extracted following the above procedure and the resulting organic extract (14.1 g) was submitted to dereplication by using an ultra-high-performance liquid chromatography/high-resolution mass spectrometry (UHPLC-HRMS) on a Q Exactive Focus mass spectrometer coupled to an UltiMate 3000 UHPLC with Xcalibur software v.4.0.27.19 (Thermo Fisher Scientific). External calibration was performed using the LTQ VELOS ESI Positive Ion Calibration Solution and Negative Ion Calibration Solution (ref. 11360360). The raw MS was analyzed using Compound Discoverer software v2.1 (all equipment, software and calibration solutions from Thermo Fisher Scientific). Liquid chromatography. The separation was performed using a Waters XBridge column C18, 2.1 × 150 mm, 3.5 μm particle size, P/N 186,003,023 (Optima™ LC-MS Grade, Thermo Fisher Scientific). The column temperature was maintained at 30 °C and the mobile phase consisted of: (A) H_2_O with 0.1% formic acid (V/V) and (B) CH_3_CN with 0.1% formic acid (V/V). It started with a 13 min of linear gradient, from 1% to 99% of B, followed by a 2 min isocratic phase at 99% of B, then a linear gradient from 99% to 1% of B by 1 min and a 4 min isocratic at 99% of A, with a flow rate of 0.5 mL/min. The exact mass of the components was compared against the Antimarin^®^ and SciFinder^®^ database platform. If a plausible match was found, considering the exact mass/molecular formula, the molecule was considered as a putative component of the fraction.

### Microalgae Strains and Growth Conditions

For the microalgae growth assays, four species were used, three marine and one from fresh water. *Isochrysis galbana* (microalgae bank Algoteca de Coimbra, Portugal) was grown in f/2 (Guillard’s medium for diatoms). The diatom *Phaeodactylum tricornutum* strain CCAP 1055/1, obtained from the Culture Collection of Algae and Protozoa of the Scottish Association for Marine Science (CCAP-UK), was grown in f/2 + Si medium (supplemented with silica and with a corrected concentration of sodium nitrate of 1.18 mM). They were grown in 100 mL Erlenmeyer flasks at 19 °C, in a rotary shaker at 130 rpm (Innova™ 2100, New Brunswick Scientific). The green microalgae *Haematococcus pluviali*s strain CCAP 34/6 (CCAP-UK) and *Tetraselmis chuii* (Algoteca de Coimbra, Portugal) were grown in 3 N-BBM + V (Bold Basal Medium with 3-fold Nitrogen and Vitamins) and f/2 medium, respectively, on a rotary shaker at 23 °C, 110 rpm (Agitorb 200IC, Aralab). All microalgae were cultivated under a photosynthetically active photon flux density of 30 µmol photons.m^− 2^.s^− 1^ with a 16 h light/8 h dark cycle (Afonso et al. 2022).

### Evaluation of Bioactive Marine Extracts

An amount of approximately 1 mg of each of the aforementioned marine organic extracts was accurately weighed for subsequent analysis. A volume of 10 µL of dimethyl sulfoxide (DMSO) was added to the crude extracts, vortexed for less than 1 min, and the process repeated until all extracts were soluble. This resulted in a final stock concentration of 10 mg/mL. Our experimental methodology incorporated initial DMSO testing, where we concluded that a 0.25% (V/V) of DMSO did not significantly affect cell growth. Assays were performed in a 96 well-plate and the working volume was 200 µL in each well. Cells were inoculated with the 65 marine organic extracts to a final concentration of 0.025 mg/mL, at the beginning of the lag phase, which corresponds to day 1 of growth. Microalgae were grown for 9 days in an incubator (Agitorb 200IC) at 23 °C and 130 rpm. On the selected days of the microalgae growth (0, 2, 5, 7, 8 or 9), the optical density (OD) at 600 nm was measured in a microplate spectrophotometer (Epoch biotek) and normalized for day 0 to account for minor differences observed in the initial inoculum. Before each measurement, cells were resuspended with a multi-channel pipette.

Spectrophotometric analyses were performed using three technical replicates for each assay. Growth inhibition percentage was calculated according to the following equation (Eq. 1). The inhibition percentage was calculated on day 5 (Haris et al. 2022; Shahneh et al. 2013).

1 $$\%\;Inhibition=100-\left(\frac{Test\;OD}{Non-treated\;OD}\right)\times100$$

Data were analyzed using GraphPad Prism 9.5.1 software and are presented as a single value for one biological sample (*n* = 1) or as the mean ± standard deviation (SD) for two to five biological replicates.

For the fractions and isolated compounds of *H.* (*Rhizoniera*) *curacaoensis* (EY18-4), the assays were replicated under identical conditions, and the data were analyzed using nonlinear regression, applying a fourth-order polynomial equation to model the results. Additionally, the Log*P* values of the isolated compounds were calculated using the tools provided in ChemDraw Professional (v23.0.1) (Zorrilla et al. 2024).

## Results and Discussion

### Screening of Marine Organic Extracts for Potential Algal Growth Regulation Effect

Specimens were collected by snorkeling and SCUBA diving in different coastal areas of the Yucatán Peninsula, Mexico. Each specimen was assigned a unique code based on its specific geographic origin. The full list of 65 marine species is shown in Table S.1. The marine organisms were extracted with a mixture of dichloromethane–methanol to give, after removal of the solvent, the corresponding organic extracts. In this study, we aimed to evaluate the cytotoxic effects of compounds produced by marine organisms on microalgae in search of algicidal agents to mitigate HABs. Additionally, we sought to find compounds that might stimulate microalgae growth resulting in increased biomass yields, as they could serve as promoters of algae growth for industrial applications.

In the initial screening, all 65 specimens were evaluated for their potential anti-algal effects against a set of four target microalgae. The selection criteria for the target microalgae aimed to comprise both green and brown microalgae, representing diverse marine and freshwater environments. For this purpose, we selected *P. tricornutum*, *I. galbana*, *H. pluvialis* and *T. chuii.* Extracts were added on day 1 of cultivation and microalgae OD was monitored for 8 consecutive days. Day 5 was selected to study the cytotoxic or growth promotion effects exhibited by each extract. To ensure accuracy, OD values were normalized to account for minor differences observed in the initial inoculum. It is important to note that, for some extracts, the data represented a single biological sample (*n* = 1) with technical replicates. Therefore, these results should be interpreted as preliminary screening data, identifying candidates for further validation rather than definitive evidence of algicidal or growth-promoting activity. The inhibition percentage was calculated for each extract and visual observations corresponded well with the quantitative results obtained.

**Fig. 1:**
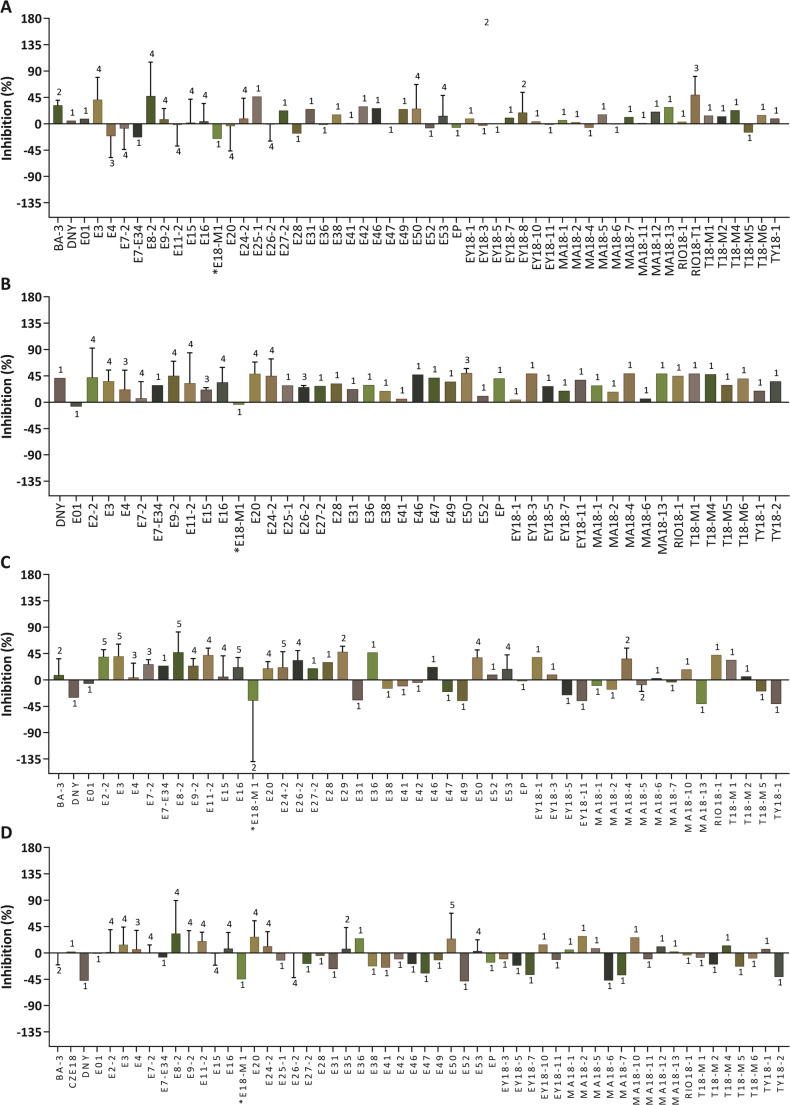
Growth inhibition (< 50% inhibition) and growth promotion (< 50% promotion) percentages of microalgae species: (**A**) *Phaeodactylum tricornutum*, (**B**) *Isochrysis galbana*, (**C**) *Haematococcus pluvialis* and (**D**) *Tetraselmis chuii* when incubated with 0.025 mg/mL of different marine organism extracts. Each column in the graph represents the mean inhibition, while the error bars indicate the standard deviation. Numbers associated with each column indicate the number of biological samples. Each biological sample consisted of three technical replicates. The asterisk symbol marks the two species chosen for further investigation. Codes and species names as listed in the legend: BA-3: *Briareum asbestinum*, CZE18: *Myrmekioderma gyroderma*, DNY: *Scopalina ruetzleri*, E01: *Didemnum* sp., E2-2: *Leucetta floridana*, E3: *Plakinastrella onkodes*, E4: *Melophlus hajdui*, E7-2: *Trididemnum solidum*, E7-E34: *Clathria virgultosa*, E8-2: *Didemnum perlucidum*, E9-2: *Ircinia felix*, E11-2: *Spongia tubulifera*, E15: *Niphates digitalis*, E16: *Callyspongia vaginalis*, E18-M1: *Halichondria melanadocia*, E20: *Tethya* sp., E24-2: *Ircinia strobilina*, E25-1: *Agelas dilatata*, E26-2: *Agelas sceptrum*, E27-2: *Agelas clathrodes*, E28: *Callyspongia longissima*, E29: *Amphimedon compressa*, E31: *Callyspongia plicifera*, E35: *Monanchora arbuscula*, E36: *Aplysina cauliformis*, E38: *Aaptos* sp., E41: *Polycarpa* sp., E42: *Aplysina fulva*, E46: *Aplysina fistularis*, E47: *Aplysina muricyana*, E49: *Niphates erecta*, E50: *Aiolochroia crassa*, E52: *Ircinia strobilina*, E53: *Scopalina ruetzleri*, EP: *Xestospongia muta*, EY18-1: *Cliona delitrix*, EY18-3: *Cliona varians*, EY18-5: *Aplysina fulva*, EY18-7: *Scopalina ruetzleri*, EY18-8: *Polysyncraton* sp., EY18-10: *Clathrina* sp., EY18-11: *Clathria gomezae*, MA18-1: *Mycale laevis*, MA18-2: *Cinachyrella kuekenthali*, MA18-4: *Aiolochroia crassa*, MA18-5: *Mycale laevis*, MA18-6: *Chondrilla caribensis f. hermatypica*, MA18-7: *Niphates erecta*, MA18-10: *Agelas clathrodes*, MA18-11: *Ircinia felix*, MA18-12: *Niphates erecta*, MA18-13: *Ectyoplasia* sp., RIO18-1: *Chondrilla* sp., RIO18-T1: *Eudistoma amanitum*, T18-M1: *Clavelina* sp., T18-M2: *Ecteinascidia* sp., T18-M4: *Didemnum* sp., T18-M5: *Polyclinum* sp., T18-M6: *Molgula* sp., TY18-1: *Phallusia nigra*, TY18-2: *Eudistoma* sp.

**Fig. 2:**
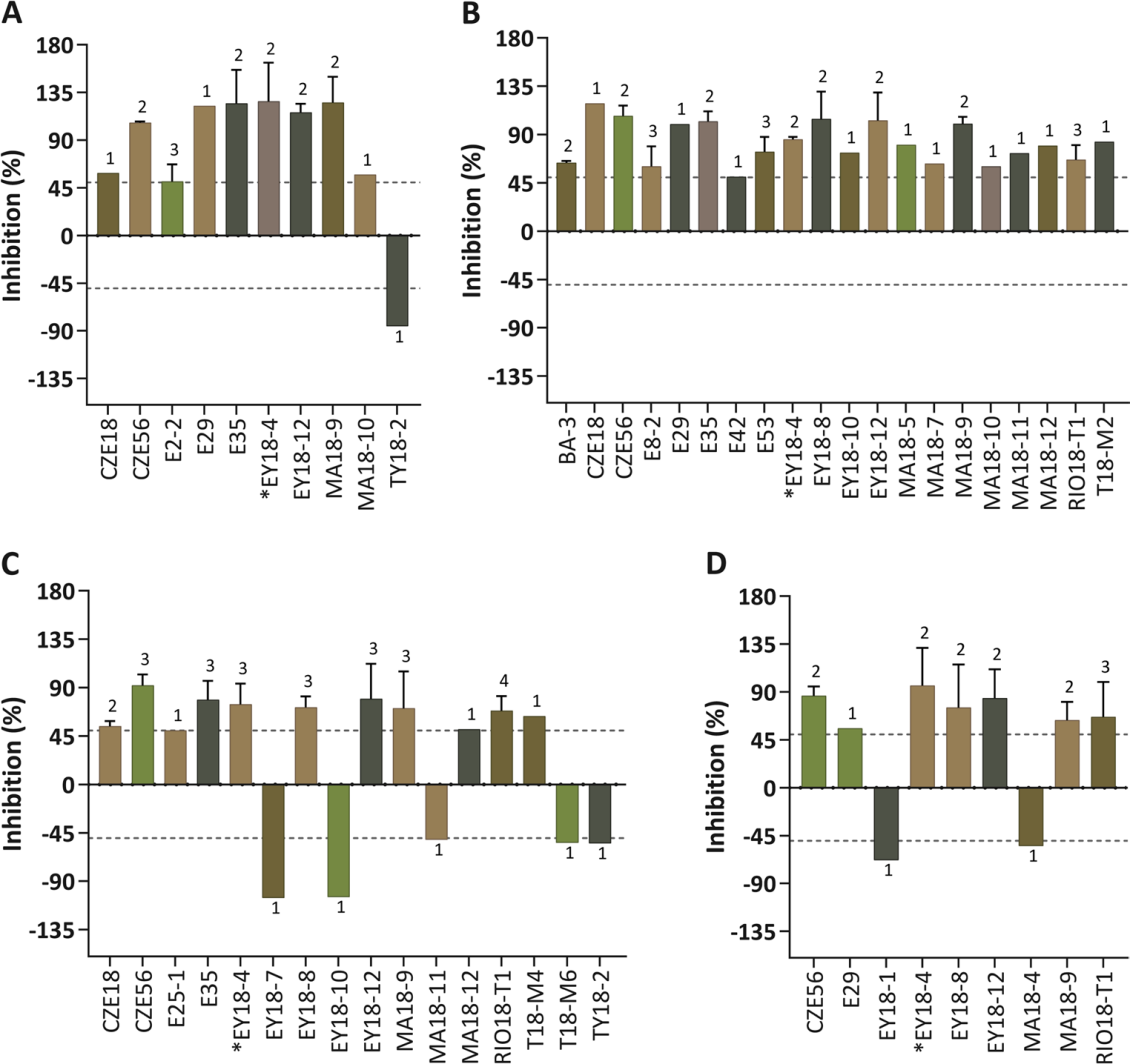
Growth inhibition (> 50% inhibition) and growth promotion (< -50% inhibition) data for (**A**) *Phaeodactylum tricornutum*, (**B**) *Isochrysis galbana*, (**C**) *Haematococcus pluvialis* and (**D**) *Tetraselmis chuii*. Dashed lines delineate the thresholds for growth inhibition and growth promotion. Numbers associated with each column represent the number of biological samples. The asterisk symbol marks the one selected species. Codes and species names as listed in the legend: BA-3: *Briareum asbestinum*, CZE18: *Myrmekioderma gyroderma*, CZE56: *Agelas citrina*, E2-2: *Leucetta floridana*, E8-2: *Didemnum perlucidum*, E25-1: *Agelas dilatata*, E29: *Amphimedon compressa*, E35: *Monanchora arbuscula*, E42: *Aplysina fulva*, E53: *Scopalina ruetzleri*, EY18-1: *Cliona delitrix*, EY18-4: *Haliclona (Rhizoniera) curacaoensis*, EY18-7: *Scopalina ruetzleri*, EY18-8: *Polysyncraton* sp., EY18-10: *Clathrina* sp., EY18-12: *Dysidea* sp., MA18-4: *Aiolochroia crassa*, MA18-5: *Mycale laevis*, MA18-7: *Niphates erecta*, MA18-9: *Ectyoplasia ferox*, MA18-10: *Agelas clathrodes*, MA18-11: *Ircinia felix*, MA18-12: *Niphates erecta*, RIO18-T1: *Eudistoma amanitum*, T18-M2: *Ecteinascidia* sp., T18-M6: *Molgula* sp., TY18-2: *Eudistoma* sp

In the initial screening of 65 marine extracts, microalgae growth was evaluated after 5 days of incubation and was assessed against the non-treated cells for each microalgal species individually. Extracts were divided into two categories based on their activity. Extracts showing moderate effects (< 50% inhibition or > − 50% growth promotion) are presented in Fig. 1, while extracts exhibiting strong activity (> 50% inhibition or < − 50% growth promotion) are highlighted in Fig. 2. Out of the 65 extracts tested, there was a stronger inhibitory effect observed in *I. galbana* compared to *H. pluvialis*. This observation aligns with existing research suggesting that *H. pluvialis*, with its capacity for astaxanthin production, may exhibit greater resilience to environmental stressors (Oslan et al. 2021; Park et al. 2023). Among the 65 tested extracts, *Agelas citrina* (CZE56), *Monanchora arbuscula* (E35), *Haliclona (Rhizoniera) curacaoensis* (EY18-4), *Dysidea sp.* (EY18-12), *Ectyoplasia ferox* (MA18-9), and *Eudistoma amanitum* (RIO18-T1) exhibited pronounced growth inhibitory activity against the four selected microalgal species. These extracts inhibited microalgae growth by at least 50%.

Sponges of the genus *Haliclona* are renowned for their prolific production of diverse secondary metabolites, with bioactive alkaloids being the most prevalent. *Haliclona dancoi* exhibited a chemical defense mechanism through polar extracts, demonstrating algicidal properties by effectively eliminating diatoms. This observation underscored the broad spectrum of compounds generated by *Haliclona* sponges, some of which hold potential applications as algicides (Amsler et al. 2000). Contrastingly, extracts, such as *Eudistoma sp*. (TY18-2), *Cliona delitrix* (EY18-1), *Scopalina ruetzleri* (EY18-7), and *Clathrina* sp. (EY18-10) facilitated the growth of *P. tricornutum*, *T. chuii*, and *H. pluvialis*, respectively, while *Halichondria melanadocia* (E18-M1) showed preliminary growth-promoting effects in all four species. This might suggest a mutualistic relationship between algae and sponges. For example, the macroalgae *Jania adhaerens* relies on the sponge *Haliclona caerulea* for survival in deeper sea areas (Enríquez et al. 2009). Sponge tissues revealed a much greater efficiency to neutralize both peroxyl and hydroxyl radicals during the period of elevated symbiotic association (Regoli et al. 2004). This highlights the potential symbiotic association between algae and sponges, where each organism benefits from the presence of the other (Hustus et al. 2023). The effects of the tested extracts were not always consistent with the microalgal species. For instance, the extract *Melophlus hajdui* (E4) showed algicidal activity against *I. galbana* but promoted the growth of *P. tricornutum.* Our observations revealed that certain extracts exhibited a strong inhibition of microalgae growth (> 50%), while others demonstrated a clear enhancement, reaching a minimum of 50% growth (Fig. 2). This variability underscores the diverse and sometimes opposing influences of these extracts on microalgal growth. It was considered that when the growth inhibition percentage exceeded 50%, the extract demonstrated inhibitory effects against the microalgae. Conversely, if the percentage fell below − 50%, the extract promoted microalgae growth (Shahneh et al. 2013).

Some extracts demonstrated varying results across different assays on microalgal growth, highlighting the complexity and variability intrinsic to living organisms. While microalgae growth remained generally consistent from batch to batch we observed inherent biological variability, potentially influencing the outcomes, but also from the limited number of biological replicates, which reduces the ability to generalize these findings. Additionally, the complexity of the marine organism extracts, containing multiple compounds, made it challenging to ensure uniformity between assays.

### Selection of the Most Promising Extracts and Posterior Chemical Analysis

After a comprehensive analysis, extract EY18-4 (*Haliclona* (*Rhizoniera*) *curacaoensis*) emerged as a promising candidate for subsequent studies. This extract consistently demonstrated substantial cytotoxic effects across all four microalgae species, also validated through visual inspections. After an 8-day observation period, micro-plate wells containing microalgae treated with EY18-4 extract exhibited a strikingly transparent appearance, markedly different from control wells lacking the marine extract (control). These microalgae, being photosynthetic organisms continuously producing chlorophyll and pigments, typically exhibit distinct pigmentation – brown hues for *P. tricornutum* and *I. galbana*, and green for *H. pluvialis* and *T. chuii.* The distinct coloration typically associated with microalgae was notably absent in the samples treated with the extract.

In addition to the promising results observed with extract EY18-4, another intriguing observation emerged during the analysis – the potential positive effects of extract E18-M1 (*Halichondria melanadocia*) on microalgae growth (Fig. 1). In contrast to the cytotoxic effects observed with EY18-4, E18-M1 exhibited an apparent growth-promoting effect of the microalgae species under investigation, with the most pronounced response observed in *H. pluvialis* (Fig. 1C). This was supported by visual inspections over the 8-day cultivation period, which revealed an increased culture density compared to the control. These observations highlight the multifaceted nature of sponge-derived extracts and underscore their potential applications in various fields, including biotechnology, environmental remediation, and pharmaceuticals. For this reason, further exploration of EY18-4 and E18-M1 organic extracts was conducted to identify or isolate the potential compounds responsible for the observed biological activity.

### Identification of the Algicidal Compounds from *Haliclona (Rhizoniera) curacaoensis* Marine Sponge Extract (EY18-4)

The EY18-4 (7.9 g) extract was submitted to successive liquid-liquid extraction involving water, *n*-hexane, dichloromethane, methanol, and *n*-butanol, following the fractionation methodology (Pech-Puch et al. 2023), to yield the WW, FH, FD, FM, and WB fractions after solvent removal (Table S.2). Subsequently, these fractions were added to the microalgae, as described previously. To maintain consistency and ensure comparable testing conditions, we decided to utilize the same final concentration for these fractions. This decision was based on the absence of precise information regarding the proportion of each fraction in the original sponge extract. By maintaining an identical final concentration, it aimed to standardize the testing conditions across all fractions for a more reliable comparative analysis.

**Fig. 3:**
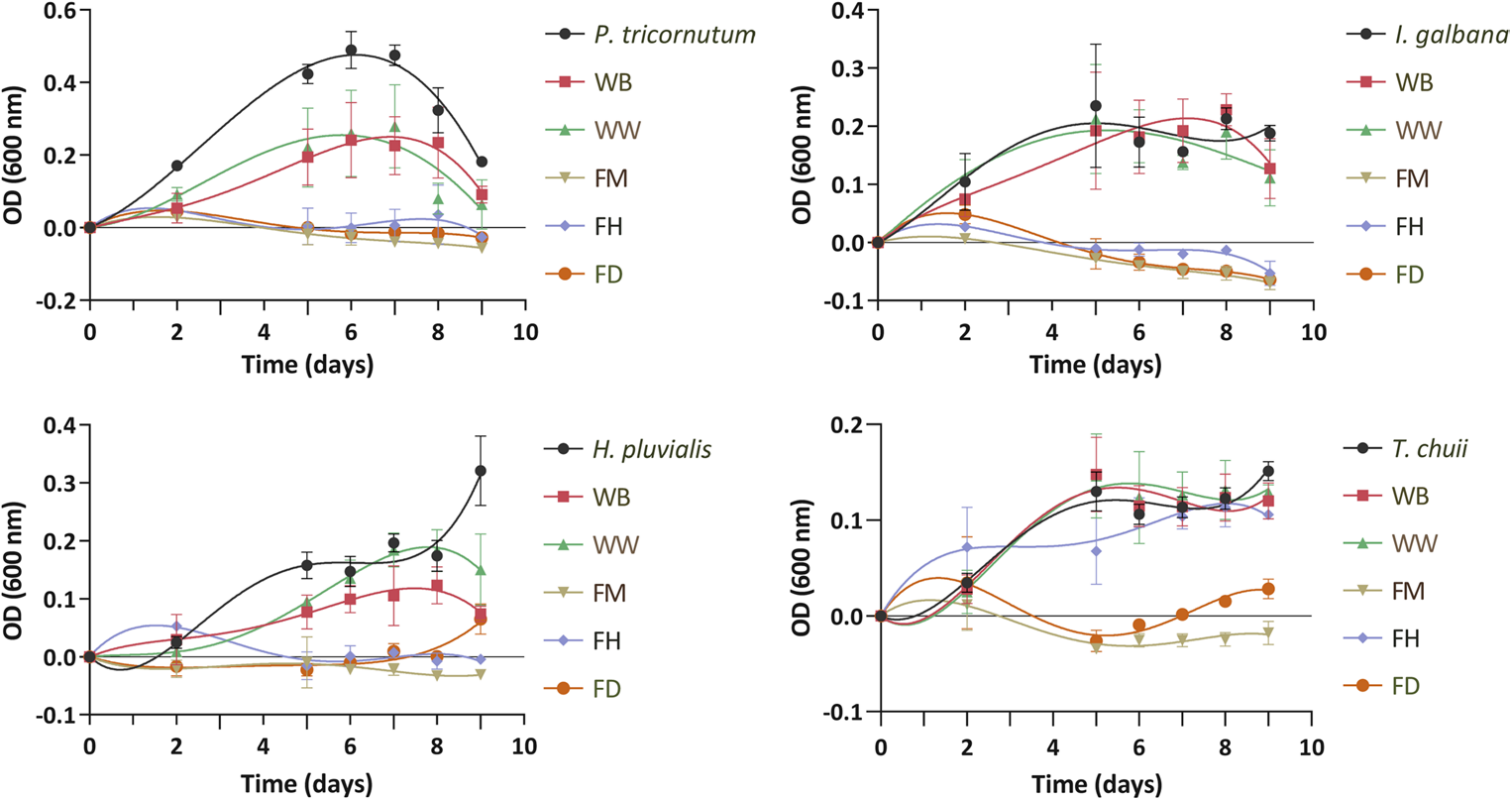
Evaluation of microalgae growth following the addition of 0.025 mg/mL of *n*-butanol (WB), water (WW), methanol (FM), *n*-hexane (FH), and dichloromethane (FD) fractions obtained from the organic extract (EY18-4) of *Haliclona (Rhizoniera) curacaoensis* by measuring optical density (OD) at 600 nm across the growth curve. Lines represent trend adjustments, in the form of polynomial equations. Data is represented as mean OD values, with error bars indicating the standard deviation (*n* = 3 technical replicates)

Autotrophic growth of *P. tricornutum*, *I. galbana*, *H. pluvialis*, and *T. chuii* was observed over 9 days under control conditions (with no extract addition) and in the presence of five distinct fractions obtained from the EY18-4 extract. The OD data, represented in Fig. 3, was plotted and depicted using polynomial lines to highlight trends and patterns over the experimental duration. The FD and FM fractions from the EY18-4 extract exhibited promising outcomes during this assay. Over a 9-day experimental period, the impact of these fractions on microalgae growth was particularly striking. For all four microalgal species tested, the incubation with fractions FD and FM resulted in almost negligible growth by the end of the incubation period. This substantial inhibitory effect indicated a potent suppression of cell proliferation, indicating these fractions as particularly impactful in impeding microalgae growth. Fraction FD and FM were chosen as candidates for further purification and testing, due to their potent inhibitory effect on microalgae growth. This can be a potentially promising avenue for identifying and isolating active compounds with potent algicidal properties. In fact, compared with the number of algicidal bacterial strains that have been isolated and identified, there are fewer algicidal compounds successfully extracted, purified, and identified (Gallardo-Rodríguez et al. 2019; Gupta 2019).

**Fig. 4:**
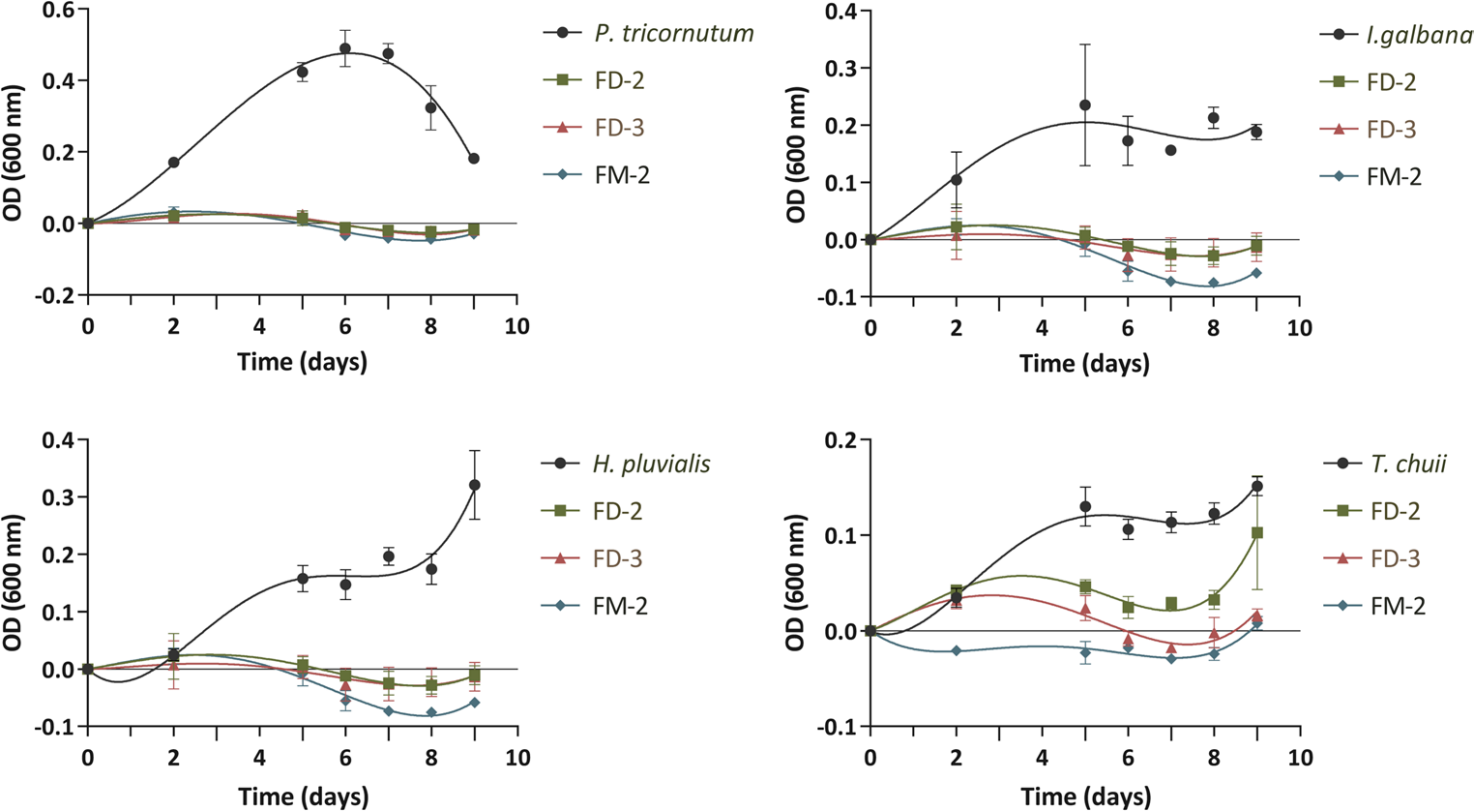
Evaluation of microalgae growth following the addition of 0.025 mg/mL of FD-2, FD-3 and FM-2 subfractions obtained from the SPE of the FD and FM fractions of the *Haliclona (Rhizoniera) curacaoensis* organic extract (EY18-4). Trend adjustments are illustrated by lines in the form of polynomial equations. Data is represented as mean OD values, with error bars representing the standard deviation (*n* = 3 technical replicates)

The FD and FM fractions submitted to SPE were eluted with water-methanol as mobile phase to afford FD (1–6) and FM (1–6) subfractions, respectively. The subfractions FM-2, FD-2, and FD-3 were added to the microalgae as previously described. The assay depicted in Fig. 4 revealed that FM-2 and FD-3 subfractions exhibited a significant cytotoxic effect, completely inhibiting microalgae proliferation upon incubation. In contrast, fraction FD-2 displayed a relatively minor reduction in OD values in *T. chuii* when compared to FM-2 and FD-3, indicating a diminished impact on growth on a preliminary analysis. Since these subfractions still represent mixtures of compounds, further separation was necessary in order to identify the individual constituents responsible for the observed algicidal activity. For this reason, FM-2 and FD-3 subfractions were further separated by HPLC.

### Isolation of Pure Compounds of EY18-4 *Haliclona (Rhizoniera) curacaoensis* Marine Sponge Extract

**Fig. 5:**
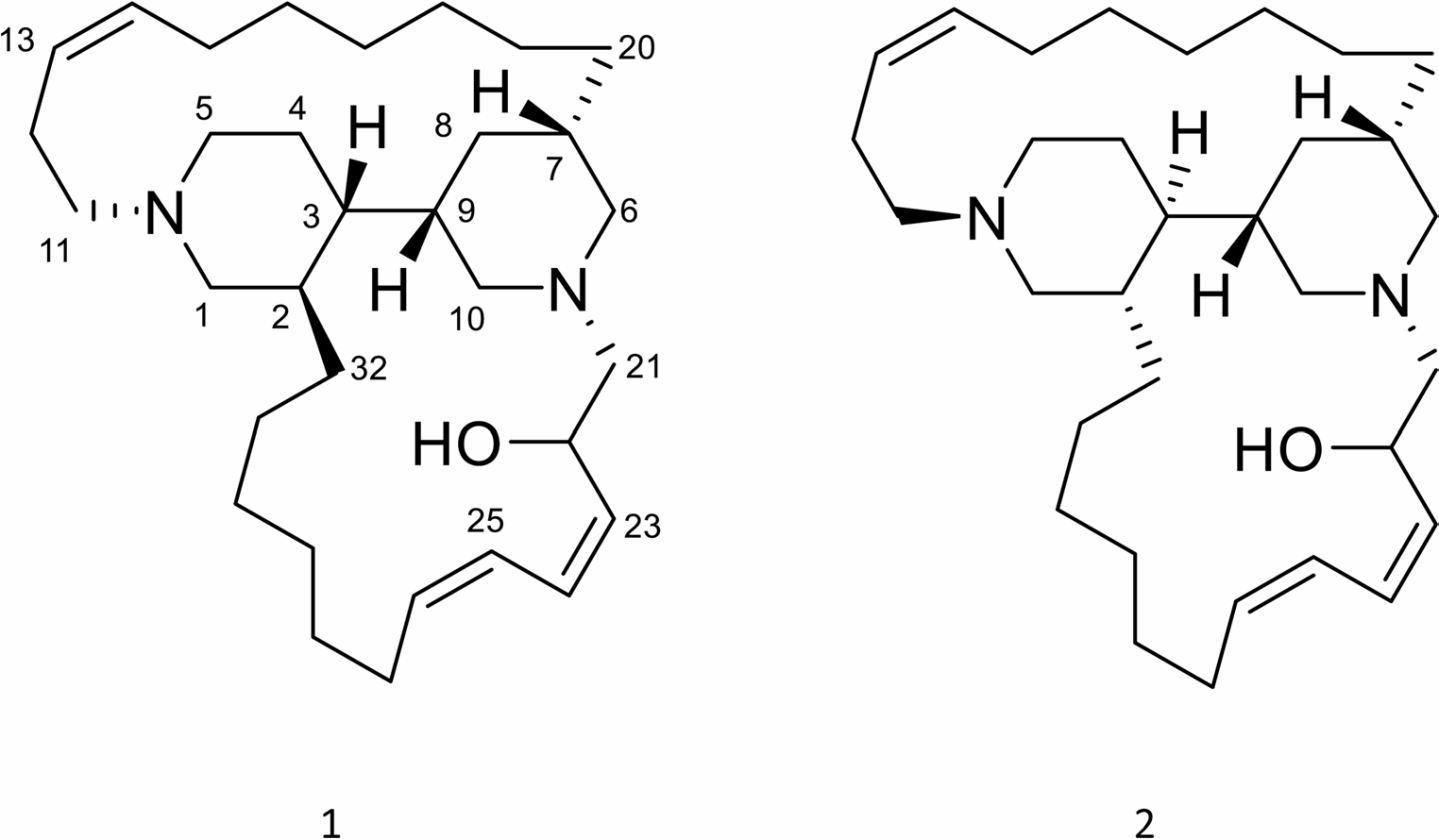
Chemical structures of arenosclerin A (**1**) and arenosclerin C (**2**) isolated from *Haliclona* (*Rhizoniera*) *curacaoensis*, *extract (EY18-4)*

The bioactive subfraction FM-2 was separated by HPLC to give the HPLC fraction FM-2-H2 eluted with a Rt of 9.5 min (see HPLC chromatogram in Figure S.1) which afforded, after evaporation of the solvent, compound **1**. The HPLC fraction FD-3-H1 eluted with a Rt of 16.7 min from the HPLC separation (see HPLC chromatogram in Figure S.2) of the bioactive FD-3 subfraction gave, after evaporation of the solvent, compound **2** (Fig. 5).

The molecular formula of **1** isolated as a yellow solid [α]^25^_D_ = -24.2 º (c 0.14, MeOH) was established from its HRESIMS, which showed a [M + H]^+^ ion at *m/z* 483.4302 (calculated for C_32_H_55_N_2_O, *m/z* 422.4312, Δ = 2.43 ppm), indicating seven degrees of unsaturation. The ^1^H and ^13^C NMR spectral data of 1 in CD_3_OD (see Figure S.3 and S.4, Table S.3) were indicative of a haliclonacyclamine/arenosclerin-type tetracyclic alkaloid compound. Characteristic signals of six double bond methines at *δ*_H_/*δ*_C_ 5.61 (1H, m, H14)/136.4 (C14) and 5.44 (1H, dd, *J =* 7.6, 8.0 Hz, H13)/123.3 (C13); 6.56 (1H, t, *J =* 11 Hz, H24)/127.5 (C24) and 5.55 (1H, m, H23)/133.1 (C23); 6.44 (1H, t, *J =* 11.1 Hz, H25)/124.9 (C25) and 5.69 (1H, m, H26 )/137.3 (C26), along with two *N*-methylene pairs at δ_H_/δ_C_ 3.39 (1H, m, H1a) and 2.99 (1H, m, H1b)/52.5 (C1), 3.37 (2H, m, H5)/50.6 (C5), 3.33 (1H, m, H6a ) and 2.56 (1H, dd, *J =* 12.0, 12.0 Hz, H6b)/59.3 (C6) and 3.42 (1H, m, H10a) and 3.01 (1H, m, H10b)/59.5 (C10), corresponding to the bispiperidine system, suggested that 1 was a macrocycle with conjoint piperidine alkaloid rings. Three degrees of unsaturation were assigned to three double bonds in 1 from the six sp^2^ carbons displayed in its ^13^C-NMR DEPT-135 spectrum, while the remaining four unsaturation degrees were ascribed to a tetracyclic carbon skeleton. The presence of an allylic hydroxyl group was evident based on the chemical shift of the carbinol methine at *δ*_H_/*δ*_C_ 5.01 (1H, br t, *J* = 8.2 Hz, H22)/62.4 (C22). Finally, the comparison of ^1^H and ^13^C NMR data of 1 with those for haliclonacyclamine/arenosclerin structures reported in the literature allowed us to identify compound 1 as arenosclerin A, the tetracyclic alkaloid isolated from *Arenosclera brasiliensis* sponge collected in Brazil (Torres et al. 2000).

The molecular formula of **2**, isolated as a brown solid [α]^25^_D_ = -44.3 (c 0.11 MeOH), was established based on the [M + H]^+^ peak at *m/z* 483.4330, observed in its HRESIMS (calculated for C_32_H_55_N_2_O, *m/z* 422.4312, Δ = 3.30 ppm), with seven unsaturation degrees. The similarity of ^1^H and ^13^C NMR spectral data in CDCl _3_ and CD_3_OD of **2** (see Figures S.5 to S.10, Table S.4 and S.5) to those of 1 suggested that **2** was also a hydroxylated macrocycle with conjoint piperidine alkaloid rings, and they must be diastereoisomers. The ^1^H and ^13^C NMR spectral data of **2** were found to be identical with those reported for arenosclerin C (Fig. 5), a hydroxylated alkaloid also isolated from *A. brasiliensis* (Torres et al. 2000). Among the alkylpiperidine alkaloids, the isolation of various stereoisomers with identical planar structures has been documented. Arenosclerins C was also isolated from the sponge *Arenosclera brasiliensis* by Torres et al. (2000).

**Fig. 6:**
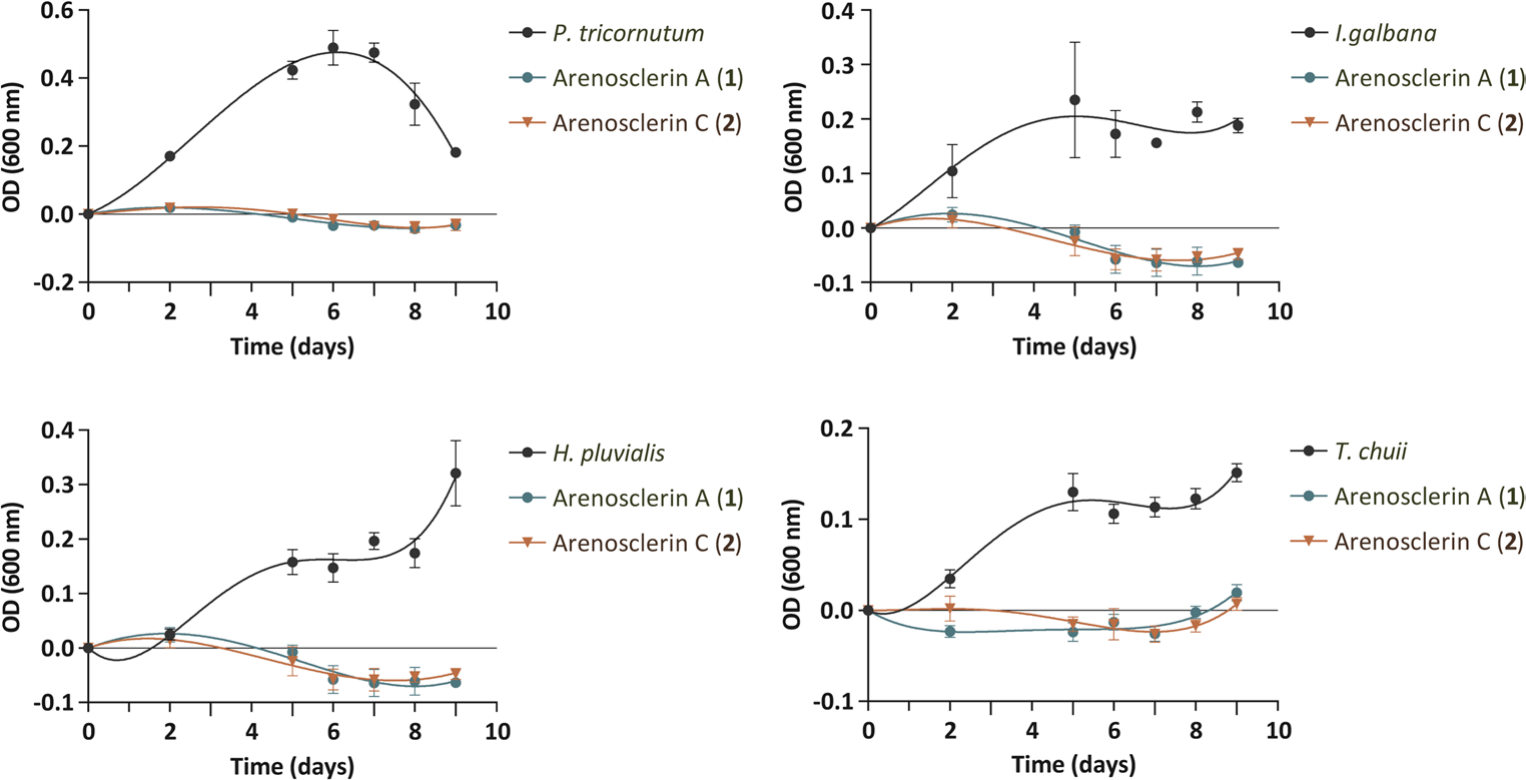
Assessment of microalgae growth following the addition of 0.025 mg/mL of arenosclerin A (**1**) and arenosclerin C (**2**) isolated from *Haliclona* (*Rhizoniera*) *curacaoensis* extract (EY18-4). Trend adjustments are illustrated by lines in the form of polynomial equations. Data is represented as mean OD values, with error bars representing the standard deviation (*n* = 3 technical replicates)

As described previously, the aqueous methanolic and dichloromethane fractions underwent SPE and RP-HPLC, resulting in the isolation of arenosclerin A (**1**) and arenosclerin C (**2**) with retention times of 14.6 and 16.7 min, respectively. Since the corresponding fraction exhibited strong algicidal activity, it was important to test the isolated metabolites individually in order to clarify whether the activity could be attributed to a single compound, to both compounds acting independently, or to a synergistic interaction between them. Subsequently, each compound was added to the microalgae according to the established protocol. This assay (Fig. 6) revealed that purified compounds exhibited a significant cytotoxic effect, completely inhibiting microalgae proliferation upon incubation. Arenosclerins A and C demonstrated potent algicidal properties across all tested microalgae species, underscoring their potential as effective agents for controlling or regulating microalgae proliferation. Their calculated Log*P* (Vraka et al. 2017) of 6.8 indicates high lipophilicity, which enhances membrane permeability and facilitates their ability to reach intracellular targets. While high lipophilicity may increase the risk of nonspecific interactions and potential toxicity, the observed inhibitory effects of arenosclerins A and C on microalgae species suggest that these compounds could play an ecological role in regulating algae growth in marine environments. However, consistent with reports of antimicrobial and cytotoxic activity (Torres et al. 2002), it is possible that these alkaloids may function as general chemical defense metabolites rather than exhibiting strict target specificity. Elucidating its mechanism of action presents interesting research opportunities for optimizing its application in aquatic environments (Newman and Cragg 2020).

The study performed by Torres et al. (2002) reported antibacterial and cytotoxic activities of arenosclerins A and C, including activity against *Staphylococcus aureus*, *Pseudomonas aeruginosa*, and Methicillin-resistant *S. aureus* (MRSA), and several mammalian cancer cell lines. Therefore, while arenosclerins may have evolved to target algal competitors, their broader antimicrobial and cytotoxic effects highlight the need for further studies to determine their selectivity, ecological relevance, and mechanism of action before its application in aquatic environments.

Several alkaloids and nucleosides isolated from the marine sponge *Haliclona* sp. have been reported in the literature (Baker et al. 1988; Jaspars et al. 1994; Wang et al. 2009; Zhang et al. 2016). These compounds underwent structural characterization and subsequent testing to evaluate their potential applications, including cytotoxicity, anti-microbial and antiproliferative effects, or antiviral properties. Moreover, indole and pyrrolidine alkaloids, including 1‑(1 H‑indol‑2‑yloxy) propan‑2‑ol and (1‑hydroxyethyl)‑3,4‑diol hydrochloride, were isolated from Red Sea *Haliclona* sp. (Al-Massarani et al. 2016). They displayed weak cytotoxic activity against distinct cancer cell lines such as HepG-2, Daoy, and HeLa (Zhang et al. 2016) successfully isolated the 3-dodecyl pyridine compound, which displayed moderate cytotoxicity against tumor cell lines A549, MCF-7 and HeLa. Kim and colleagues identified novel isoquinolinequinone derivatives from *Haliclona* sp., testing them for anti-inflammatory activity in a co-culture system of human epithelial Caco-2 and THP-1 macrophages (Kim et al. 2021). Notably, one compound, *O*-demethylrenierone, exhibited robust anti-inflammatory activity, while two others demonstrated moderate effects (1,6-dimethyl-7-hydroxy-5,8-dihydroisoquinoline-5,8-dione and *N*-formyl-1,2-dihydrorenierone). Most compounds isolated from *Haliclona sp.* were evaluated for their cytotoxicity against cancer cell lines and antimicrobial activity (Bian et al. 2020). In the present study, marine extracts were tested for their effects on microalgae growth, serving both as promoters and algicidal agents. Among the isolated compounds, arenosclerin A (1) and arenosclerin C (2) exhibited exclusively algicidal activity, which is reported here for the first time. These findings suggest the potential of *Haliclona* sp. metabolites to be explored as new algicidal agents for controlling HABs.

### Dereplication Analysis of *Halichondria melanadocia* Sponge Extract (E18-M1)

**Fig. 7:**
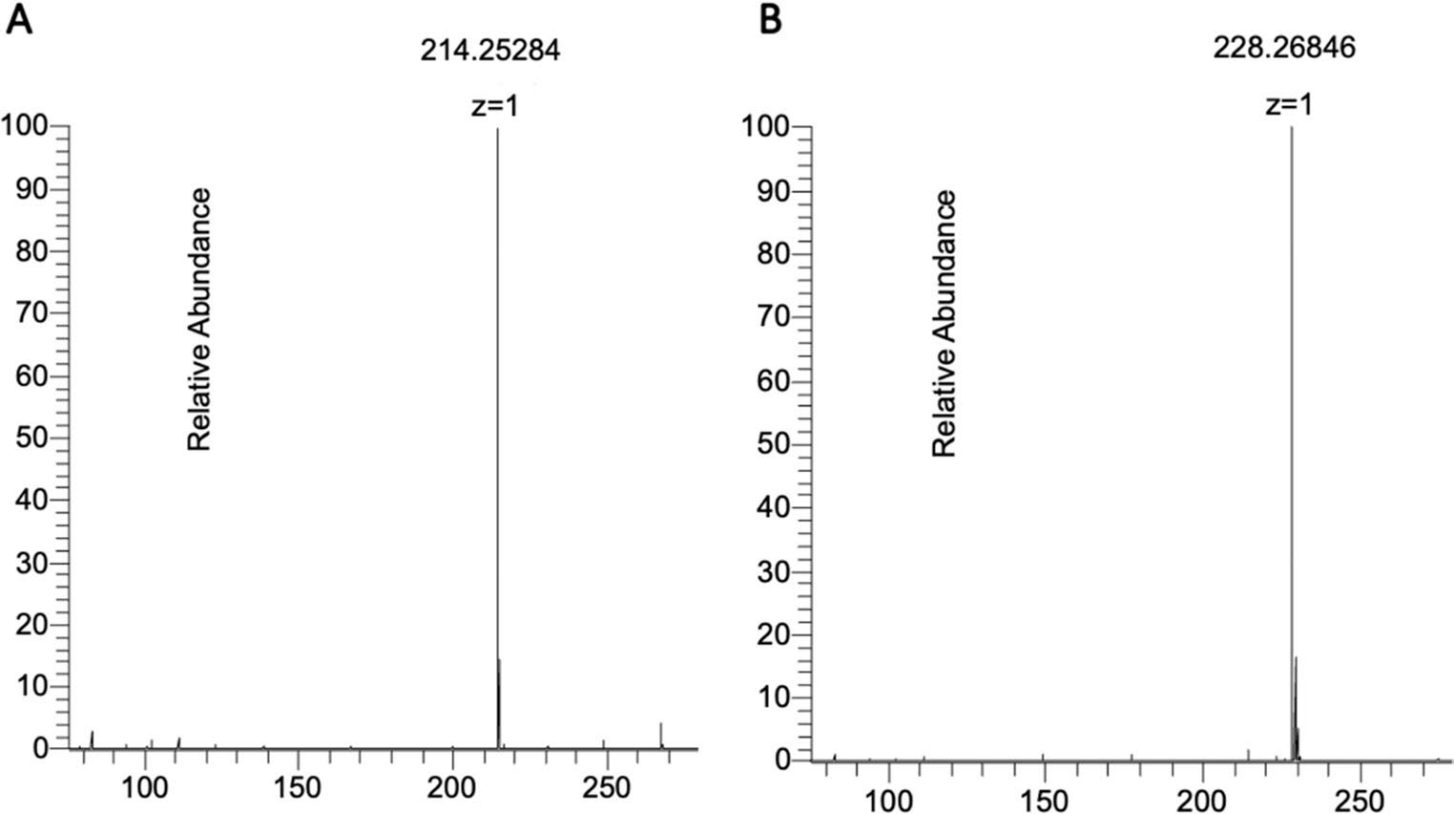
UHPLC-HRMS analysis of *Halicohondria melanadocia* extract (E18-M1). (**A**) HRMS of the chromatographic peak eluted with a retention time of 10.71, showing an [M + H]^+^ ion adduct that matches the molecular formula of C_14_H_31_N for medelamine A (3). (**B**) HRMS of the chromatographic peak eluted with a retention time of 10.81, showing an [M + H]^+^ ion adduct that matches the molecular formula of C_15_H_33_N for medelamine B (4)

To investigate the composition of the E18-M1 sponge extract that promoted microalgae growth, dereplication analysis was carried out using UHPLC-HRMS positive mode (Fig. 7, Figure S.11). The [M + H]^+^ ion adducts that corresponded to all the signals detected in the liquid chromatography–mass spectrometry (LC-MS) chromatograms were analyzed using Antimarin^®^ and Scifinder^®^ plataforms.

**Fig. 8:**
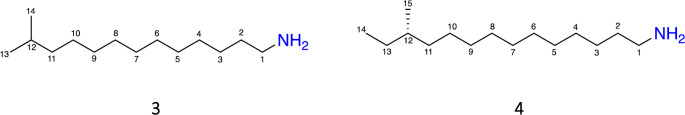
Proposed chemical structures for compounds **3** and **4** tentatively assigned as medelamine **3** and medelamine **4**, respectively, from *Halicohondria melanadocia* E18-M1 extract

A total of 16 [M + H]^+^ ion adducts that corresponded to 16 UHPLC signals were detected in the extract of E18-M1 (Table S.6). Two compounds corresponding to the [M + H]^+^ ion adducts found in the E18-M1 marine sponge extract were tentatively identified as medelamine A (3) and medelamine B (4) (Fig. 8). High-resolution mass spectrometry (HRMS) analysis of the compounds eluted with a retention time of 10.71 min revealed an [M + H]^+^ ion adduct at m/z 214.2528, matching that of medelamine A (3) (Morino et al. 1995) (calculated as m/z 214.2529, Fig. 7A). Similarly, the other compound, with a retention time of 10.81 min and an experimental value of [M + H]^+^ m/z 228.2684, corresponded to medelamine B (4) (Morino et al. 1995) (calculated as m/z 228.2685, Fig. 7B). The molecular formula C₁₄H31N assigned to the ion detected at m/z 214.2528 ([M + H]⁺) displayed a mass error of 0.47 ppm. Its corresponding M + 1 isotopic contribution of 15.1% agreed with the expected theoretical value for a 14-carbon compound. Furthermore, the neutral formula (C₁₄H_31_N) presented a DBE of zero, which was consistent with the fully saturated aliphatic amine structure of medelamine A (3). Similarly, the related metabolite detected at m/z 228.2684 ([M + H]⁺), assigned to the molecular formula C₁₅H₃₄N, exhibited a mass error of 0.44 ppm. Again, the experimental M + 1 isotopic relative abundance (17.33%) was consistent with the theoretical value expected for a 15-carbon molecule. Moreover, its corresponding neutral formula (C₁₅H₃₃N) exhibited a double bond equivalent (DBE) of zero, which agreed with the fully saturated aliphatic amine structure of medelamine B (4). The coherent variation in high-resolution mass spectrometry values, isotopic pattern, and unsaturation supports that both compounds belong to the same structural family and validates their dereplication. Further analysis is required to corroborate the proposed structures for compounds **3** and **4**, as the current set of HRMS data is insufficient for this purpose.

The identification of the remaining [M + H]^+^ ion adducts proved elusive; they may represent compounds not yet cataloged in the Antimarin^®^ and Scifinder^®^ platforms awaiting for discovery.

There is limited information available in the literature regarding medelamine A (3) and B (4). Originally, they were isolated from one culture of *Streptomyces* sp. NK14819 strain, which was collected from the soil in Saitama Prefecture, Japan (Morino et al. 1995). Morino and colleagues evaluated the cytotoxic effect of these medelamines in cancer cells, but no reports were found concerning their interaction with microalgae. Since medelamine A (3) and B (4) were present in higher concentrations in the E18-M1 extract, they could be the compounds responsible for its growth-promoting effect. Regarding the extract of the sponge *H. melanadocia* collected in the Yucatán Peninsula, its antifungal, antiproliferative, antibacterial and antiviral activity had previously been evaluated, however it did not show significant activity in any of the above (Pech-Puch et al. 2020b, 2023, 2020a). To the best of our knowledge, only three secondary metabolites have been isolated from *H. melanadocia*, a cytotoxic compound called melanodocin (Gopichand and Schmitz 1979; Johnson and Bergman 2006) and two lactams. One lactam is a derivative of the indole 3-(2-(1 H-indol-3-yl)-2-oxoethyl)-5,6-dihydropyridin-2(1 H)-one, and the other is a derivative of phenol 3-(2-(4-hydroxyphenyl)-2-oxoethyl)-5,6-dihydropyridin-2(1 H)-one (Gopichand and Schmitz 1979; Johnson and Bergman 2006). Later these two lactams were isolated from the sponge *Callyspongia* sp. and its antibacterial and cytotoxic activity was evaluated, but no toxic effect was observed (Yang et al. 2013). Finally, the lactam derived from phenol was also later isolated from a culture of the fungus *Humicola grisea*, which was collected from a Brazilian soil sample (Minas Gerais, Brazil) (Andrioli et al. 2012).

The contrasting effects of the sponge extracts *Halicohondria melanadocia* and *Haliclona curacaoensis* may reflect differences in the ecological and physiological characteristics of these species. The presence of *H. melanadocia* in mangrove environments indicates its capacity to thrive in diverse and protective habitats. Mangroves act as a shield against harsh weather conditions, offering a stable environment for many marine species. This stability supports a high level of biodiversity, including numerous symbiotic relationships between sponges and other organisms (Castellanos-Pérez et al. 2020). In contrast, *H. curacaoensis* might have a more limited ecological range. We may speculate that this difference could be attributed to variations in the physiological processes of the two sponges. *H. curacaoensis* might have more specialized habitat requirements or different interactions with microorganisms, which do not promote microalgae growth. However, while the algicidal activity of *H. curacaoensis* is supported by fractionation and pure compound analyses, the growth-promoting effect of *H. melanadocia* results should be regarded as preliminary and requires further validation through dose-response studies.

To date, this is the first report demonstrating the potential growth-promoting effects of the extract of the marine sponge *H. melanadocia* on microalgal organisms. This study provides a foundation for further investigation into the marine chemical ecology behind *H. melanadocia*, its microbiome and the secondary metabolites involved in algal growth potential, which may have various biotechnological applications. Although the structures of two compounds (**3** and **4**) from the *H. melanadocia* extract were proposed, additional compounds were detected but could not be identified. Unfortunately, the low extraction yield prevented the isolation of *H. melanadocia* secondary metabolites. The literature documents the application of macroalgae or kelp waste extracts to promote microalgae growth (Rohani-Ghadikolaei et al. 2012; Vasconcelos and Leal 2008; Zheng et al. 2016). To the best of our knowledge, no study has investigated the action of sponge extracts as growth promoters for microalgae. Our report introduces the evaluation of microalgae growth-promoters derived from marine extracts from the Yucatán Peninsula.

## Conclusion

This study highlights the ecological and biotechnological importance of sponge-derived compounds. First, we demonstrated that purified arenosclerins possess unreported algicidal activity, thereby extending their previously described bioactivity profile. Second, we report that an extract enriched in medelamines promoted microalgae growth, representing new evidence of such growth-promoting effects in this context. These contrasting activities underscore the potential applications of these compounds in biotechnology and environmental remediation.

Microalgae are essential primary producers, and understanding their growth regulation is crucial to control their growth. However, the role of marine invertebrates-derived compounds in regulating microalgae growth has remained underexplored. This study investigated 65 extracts from marine invertebrates collected in the Yucatán Peninsula to assess their effects on four microalgae species. Two sponge extracts showed a considerable activity: *Haliclona (Rhizoniera) curacaoensis* (EY18-4) inhibited microalgae growth, while *Halichondria melanadocia* (E18-M1) promoted it.

Active compounds in *H. curacaoensis* included two tetracyclic alkyl piperidine alkaloids, arenosclerins A (1) and C (2), which have significant growth inhibitory effects, though their mechanism and biosynthetic pathways remain unknown. In contrast, the *H. melanadocia* extract promoted growth in all tested microalgal species and two aliphatic primary amines—medelamine A (**3**) and medelamine B (**4**)— were tentatively identified along with additional unidentified metabolites that merit further investigation.

Future research will clarify the modes of action of these compounds through dose-response and cell viability assays, metabolic pathways analysis, and gene expression profiling, while exploring their potential applications in enhancing microalgae cultivation for environmental and industrial purposes. This work also paves the way for comparative studies on the biodiversity and ecological roles of *H. curacaoensis* and *H. melanadocia*, which will be necessary to better understand their differing impacts on microalgae.

## Supplementary Information

Below is the link to the electronic supplementary material.

Supplementary Material 1 (DOCX 1.16 MB)

## Data Availability

All the data in the manuscript are obtained from included references and available upon request.
