# Supplementary material for: Bioactive Extracts from Yucatán Marine Invertebrates as Modulators of Microalgal Dynamics
Source: Mar Biotechnol (NY). 2026 May 19;28(3):86. doi: 10.1007/s10126-026-10630-5 (PMC13186800; doi:10.1007/s10126-026-10630-5)
Supplement: Supplementary file 1 — Supplementary Material 1 (DOCX 1.16 MB) [file 10126_2026_10630_MOESM1_ESM.docx]

**Bioactive Extracts from Yucatán Marine Invertebrates as Modulators of Microalgal Dynamics**

**Dawrin Pech-Puch1,2#, Rita Pires3#, Bárbara A. Rebelo3#, Diana Grilo3, Carlos Gonzáles-Salas2,**

**Susana Eunice Calva-Pérez1, Susana M. Gomes3, Harold Villegas-Hernández1, Sergio Guillén-Hernández2, Abel M. Forero4, Jaime Rodriguez4, Carlos Jiménez4 🖂, Oscar A. L.-Rojas3 🖂, Rita Abranches3 🖂**

1 Escuela Nacional de Estudios Superiores Unidad Mérida (ENES Mérida), Universidad Nacional Autónoma de México (UNAM), Carretera Mérida-Tetiz km 4.5, Tablaje Catastral No. 6998, Municipio de Ucú, Ucú CP 97357, México.

2 Departamento de Biología Marina, Universidad Autónoma de Yucatán, Km. 15.5, Carretera

Mérida-Xmatkuil, A.P. 4-116 Itzimná, Merida CP 97100, Mexico

3 Instituto de Tecnologia Química e Biológica António Xavier (ITQB NOVA), Universidade Nova de

Lisboa, 2780-157 Oeiras, Portugal

4 CICA - Centro Interdisciplinar de Química e Bioloxía, Facultade de Ciencias, Universidade daCoruña, 15071 A Coruña, Spain

# These authors contributed equally to this work

🖂 Authors to whom correspondence should be addressed

([ritaa@itqb.unl.pt](mailto:ritaa@itqb.unl.pt); [oscar.rojas@itqb.unl.pt](mailto:oscar.rojas@itqb.unl.pt); [carlos.jimenez@udc.es](mailto:carlos.jimenez@udc.es))

**Index**

[Table S.1. List of the codes assigned to marine organic extracts and their respective species and collection sites. 3](#_Toc208666936)

[Table S.2. List of the codes assigned to the fractions of the extract *Haliclona* (*Rhizoniera*) *curacaoensis* (EY18-4). 5](#_Toc208666937)

[Table S.3. ^1^H and ^13^C NMR data of arenosclerin A (1) in CD_3_OD. 6](#_Toc208666938)

[Table S.4. ^1^H and ^13^C NMR data of arenosclerin C (2) in CD_3_OD. 7](#_Toc208666939)

[Table S.5. ^1^H (500 MHz) and ^13^C (125 MHz) NMR data of arenosclerin C (2) in CDCl_3_. 8](#_Toc208666940)

[Table S.6. [M + H]^+^ ion adducts detected in UHPLC-HRMS analyses of E18-M1 marine sponge extract. 9](#_Toc208666941)

[Figure S.1. HPLC Chromatogram of subfraction FM-2, showing the HPLC peak FM-2-H2 eluted at 9.5 min, where compound 1 was isolated. 10](#_Toc208666942)

[Figure S.2. Chromatogram of subfraction FD-3, showing the HPLC peak FD-3-H1 eluted at 16.7 min, where compound 2 was isolated. 11](#_Toc208666943)

[Figure S.3. ^1^H NMR spectrum (500 MHz, CD_3_OD) of compound 1 (arenosclerin A). 12](#_Toc208666944)

[Figure S.4. ^13^C NMR spectrum (125 MHz, CD_3_OD) of compound 1 (arenosclerin A). 12](#_Toc208666945)

[Figure S.5. ^1^H NMR spectrum (300 MHz, CD_3_OD) of compound 2 (arenosclerin C). 13](#_Toc208666946)

[Figure S.6. ^13^C NMR spectrum (125 MHz, CD_3_OD) of compound 2 (arenosclerin C). 13](#_Toc208666947)

[Figure S.9. ^1^H-^1^H COSY spectrum (500 MHz, CDCl_3_) of compound 2 (arenosclerin C). 15](#_Toc208666948)

[Figure S.10. HSQC spectrum (500 MHz, CDCl_3_) of compound 2 (arenosclerin C). 15](#_Toc208666949)

[Figure S.11. UHPLC-HRMS analysis of extract E18-M1. 16](#_Toc208666950)

#

# Table S.1. List of the codes assigned to marine organic extracts and their respective species and collection sites.

| Code | Collection site | *Species* | *Extraction  yield (%)* | *Code* | Collection site | *Species* | *Extraction  yield (%)* |
| --- | --- | --- | --- | --- | --- | --- | --- |
| BA-3 | Rio Indio, Quintana Roo | *Briareum asbestinum* | *53* | *E52* | Bermejo, Quintana Roo | *Ircinia strobilina* | *91* |
| CZE18 | Cozumel, Quintana Roo | *Myrmekioderma gyroderma* | *95* | *E53* | Cozumel, Quintana Roo | *Scopalina ruetzleri* | *93* |
| CZE56 | Cozumel, Quintana Roo | *Agelas citrina* | *98* | *EP* | Alacranes Reef, Yucatán | *Xestospongia muta* | *96* |
| DNY | Rio Indio, Quintana Roo | *Scopalina ruetzleri* | *70* | *EY18-1* | Progreso, Yucatán | *Cliona delitrix* | *93* |
| E01 | Bermejo, Quintana Roo | *Didemnum sp.* | *88* | *EY18-3* | Progreso, Yucatán | *Cliona varians* | *87* |
| E2-2 | Bermejo, Quintana Roo | *Leucetta floridana* | *80* | *EY18-4* | Progreso, Yucatán | *Haliclona (Rhizoniera) curacaoensis* | *86* |
| E3 | Bermejo, Quintana Roo | *Plakinastrella onkodes* | *90* | *EY18-5* | Progreso, Yucatán | *Aplysina fulva* | *92* |
| E4 | Bermejo, Quintana Roo | *Melophlus hajdui* | *91* | *EY18-7* | Progreso, Yucatán | *Scopalina ruetzleri* | *93* |
| E7-2 | Rio Indio, Quintana Roo | *Trididemnum solidum* | *97* | *EY18-8* | Progreso, Yucatán | *Polysyncraton sp.* | *95* |
| E7-E34 | Alacranes Reef, Yucatán | *Clathria virgultosa* | *72* | *EY18-10* | Progreso, Yucatán | *Clathrina sp.* | *89* |
| E8-2 | Rio Indio, Quintana Roo | *Didemnum perlucidum* | *99* | *EY18-11* | Progreso, Yucatán | *Clathria gomezae* | *98* |
| E9-2 | Rio Indio, Quintana Roo | *Ircinia felix* | *96* | *EY18-12* | Progreso, Yucatán | *Dysidea sp.* | *98* |
| E11-2 | Rio Indio, Quintana Roo | *Spongia tubulifera* | *95* | *MA18-1* | Mahahual, Quintana Roo | *Mycale laevis* | *97* |
| E15 | Cozumel, Quintana Roo | *Niphates digitalis* | *96* | *MA18-2* | Mahahual, Quintana Roo | *Cinachyrella kuekenthali* | *79* |
| E16 | Cozumel, Quintana Roo | *Callyspongia vaginalis* | *92* | *MA18-4* | Mahahual, Quintana Roo | *Aiolochroia crassa* | *98* |
| E18-M1 | Progreso, Yucatán (Mangrove) | *Halichondria melanadocia* | *94* | *MA18-5* | Mahaual, Quintana Roo | *Mycale laevis* | *96* |
| E20 | Cozumel, Quintana Roo | *Tethya sp.* | *98* | *MA18-6* | Mahahual, Quintana Roo | *Chondrilla caribensis f. hermatypica* | *89* |
| E24-2 | Cozumel, Quintana Roo | *Ircinia strobilina* | *97* | *MA18-7* | Mahahual, Quintana Roo | *Niphates erecta* | *92* |
| E25-1 | Cozumel, Quintana Roo | *Agelas dilatata* | *56* | *MA18-9* | Mahahual, Quintana Roo | *Ectyoplasia ferox* | *90* |
| E26-2 | Cozumel, Quintana Roo | *Agelas sceptrum* | *99* | *MA18-10* | Mahahual, Quintana Roo | *Agelas clathrodes* | *83* |
| E27-2 | Cozumel, Quintana Roo | *Agelas clathrodes* | *98* | *MA18-11* | Mahahual, Quintana Roo | *Ircinia felix* | *97* |
| E28 | Alacranes Reef, Yucatán | *Callyspongia longissima* | *96* | *MA18-12* | Mahahual, Quintana Roo | *Niphates erecta* | *90* |
| E29 | Alacranes Reef, Yucatán | *Amphimedon compressa* | *94* | *MA18-13* | Mahahual, Quintana Roo | *Ectyoplasia sp.* | *74* |
| E31 | Alacranes Reef, Yucatán | *Callyspongia plicifera* | *83* | *RIO18-1* | Rio Indio, Quintana Roo | *Chondrilla sp.* | *97* |
| E35 | Alacranes Reef, Yucatán | *Monanchora arbuscula* | *95* | *RIO18-T1* | Rio Indio, Quintana Roo | *Eudistoma amanitum* | *94* |
| E36 | Alacranes Reef, Yucatán | *Aplysina cauliformis* | *92* | *T18-M1* | Progreso, Yucatán (Mangrove) | *Clavelina sp.* | *74* |
| E38 | Alacranes Reef, Yucatán | *Aaptos sp.* | *78* | *T18-M2* | Progreso, Yucatán (Mangrove) | *Ecteinascidia sp.* | *90* |
| E41 | Alacranes Reef, Yucatán | *Polycarpa sp.* | *87* | *T18-M4* | Progreso, Yucatán (Mangrove) | *Didemnum sp.* | *96* |
| E42 | Alacranes Reef, Yucatán | *Aplysina fulva* | *84* | *T18-M5* | Progreso, Yucatán (Mangrove) | *Polyclinum sp.* | *98* |
| E46 | Alacranes Reef, Yucatán | *Aplysina fistularis* | *96* | *T18-M6* | Progreso, Yucatán (Mangrove) | *Molgula sp.* | *86* |
| E47 | Alacranes Reef, Yucatán | *Aplysina muricyana* | *86* | *TY18-1* | Progreso, Yucatán | *Phallusia nigra* | *94* |
| E49 | Alacranes Reef, Yucatán | *Niphates erecta* | *85* | *TY18-2* | Progreso, Yucatán | *Eudistoma sp.* | *99* |
| E50 | Alacranes Reef, Yucatán | *Aiolochroia crassa* | *72* |  |  |  |  |

#

# Table S.2. List of the codes assigned to the fractions of the extract *Haliclona* (*Rhizoniera*) *curacaoensis* (EY18-4).

| Code | Condition |
| --- | --- |
| WB | EY18-4 extraction with *n*-butanol |
| WW | EY18-4 extraction with water |
| FM | EY18-4 extraction with methanol |
| FH | EY18-4 extraction with *n*-hexane |
| FD | EY18-4 extraction with dichloromethane |

# Table S.3. ^1^H and ^13^C NMR data of arenosclerin A (1) in CD_3_OD.

|  | **Arenosclerin A (1) (500 MHz, CD_3_OD)** | | **Reported data for arenosclerin A***  **(400 MHz, CD_3_OD)** | |
| --- | --- | --- | --- | --- |
| **Position** | **δ ^13^C** | **δ ^1^H (m, *J*, Hz)** | **δ ^13^C** | **δ ^1^H (m, *J,* Hz)** |
| **1** | 52.6 | 3.39 (m), 2.99 (m) | 52.6 | 3.39 (m), 2.99 (m) |
| **2** | 31.5 | 1.83 (m) | 31.2 | 1.82 (m) |
| **3** | 39.6 | 1.66 (m) | 39.1 | 1.63 (m) |
| **4** | 32.3 | 1.43 (m); 1.21 (m) | 32.1 | 1.41 (m); 1.21 (m) |
| **5** | 50.6 | 3.37 (m) | 50.3 | 3.37 (m) |
| **6** | 59.3 | 3.33 (m); 2.56 (dd, 12, 12) | 59.3 | 3.33 (m); 2.56 (dd, 12, 12) |
| **7** | 35.9 | 1.86 (m) | 35.6 | 1.86 (m) |
| **8** | 30.9 | 2.07 (m) | 30.6 | 2.05 (m) |
| **9** | 42.0 | 1.81 (m) | 41.8 | 1.78 (m) |
| **10** | 59.8 | 3.42 (m); 3.03 (m) | 59.6 | 3.41 (m); 3.01 (m) |
| **11** | 49.4 | 3.31 (m); 3.09 (dd, 8.4, 8.0) | 49.3 | 3.31 (m); 3.08 (dd, 8.4, 8.0) |
| **12** | 23.3 | 2.39 (m) | 23.2 | 2.39 (m) |
| **13** | 123.3 | 5.44 (dd, 7.6, 8.0) | 123 | 5.44 (dd, 7.6, 8.0) |
| **14** | 136.4 | 5.61 (m) | 136.4 | 5.61 (m) |
| **15** | 28.7 | 2.07 (m) | 28.6 | 2.07 (m) |
| **16** | 30.6 | 1.44 (m); 1.28 (m) | 30.3 | 1.44 (m); 1.28 (m) |
| **17** | 29.0 | 1.40 (m) | 29.1 | 1.40 (m) |
| **18** | 32.8 | 1.64 (m); 1.05 (m) | 32.5 | 1.64 (m); 1.05 (m) |
| **19** | 25.5 | 1.90 (m); 1.82 (m) | 25.4 | 1.90 (m); 1.82 (m) |
| **20** | 27.5 | 1.55 (m); 1.19 (m) | 27.2 | 1.55 (m); 1.19 (m) |
| **21** | 62.6 | 3.35 (m); 3.24 (m) | 62.5 | 3.35 (m); 3.24 (m) |
| **22** | 62.4 | 5.01 (br t, 8.2) | 62.4 | 5.01 (br t, 8.2) |
| **23** | 133.4 | 5.55 (m) | 133 | 5.55 (m) |
| **24** | 127.4 | 6.56 (t, 11) | 127.4 | 6.54 (t, 11) |
| **25** | 124.9 | 6.44 (t, 11) | 124.8 | 6.44 (t, 11) |
| **26** | 137.3 | 5.69 (m) | 137.3 | 5.67 (m) |
| **27** | 27.1 | 2.50 (m); 2.08 (m) | 27 | 2.50 (m); 2.09 (m) |
| **28** | 28.8 | 1.57 (m); 1.48 (m) | 28.9 | 1.56 (m); 1.48 (m) |
| **29** | 29.1 | 1.38 (m); 1.26 (m) | 29.2 | 1.38 (m); 1.26 (m) |
| **30** | 28.8 | 1.40 (m) | 28.9 | 1.40 (m) |
| **31** | 24.8 | 1.64 (m); 1.36 (m) | 24.6 | 1.64 (m); 1.36 (m) |
| **32** | 27.9 | 1.35 (m); 1.24 (m) | 27.8 | 1.35 (m); 1.24 (m) |

* Torres et al., *J. Nat. Prod.* **2000**, *63*, 1098-1105. https://doi.org/10.1021/np9905618

# Table S.4. ^1^H and ^13^C NMR data of arenosclerin C (2) in CD_3_OD.

|  | **Arenosclerin C (2)**  **(125/ 500 MHz, CD_3_OD)** | | **Reported data arenosclerin C  (100/400 MHz, CD_3_OD)*** | |
| --- | --- | --- | --- | --- |
| **Position** | **δ ^13^C** | **δ ^1^H (mult)** | **δ ^13^C** | **δ ^1^H (mult)** |
| **1** | 51.9 | 3.24 (m); 3.17 (m) | 51.9 | 3.24 (m); 3.17 (m) |
| **2** | 40.7 | 2.08 (m) | 40.6 | 2.08 (m) |
| **3** | 36.5 | 1.9 (o) | 36.5 | 1.89 (m) |
| **4** | 33.3 | 2.17 (m); 2.03 (m) | 33.3 | 2.17 (m); 2.03 (m) |
| **5** | 48.0 | 3.45 (m); 3.26 | 47.9 | 3.45 (m); 3.26 (m) |
| **6** | 59.0 | 3.32 (m); 2.56 (m) | 58.9 | 3.32 (m); 2.56 (m) |
| **7** | 36.4 | 1.9 (o) | 36.4 | 1.89 (m) |
| **8** | 36.2 | 2.32 (m); 1.21 (m) | 36.2 | 2.32 (m); 1.21 (m) |
| **9** | 42.5 | 2.15 (m) | 42.5 | 2.15 (m) |
| **10** | 60.2 | 2.89 (t, 11.5) | 60.1 | 3.30 (m); 2.98 (t, 11.5) |
| **11** | 56.2 | 3.24 (m) | 56.2 | 3.24 (m) |
| **12** | 20.5 | 2.63 (m); 2.52 (m) | 20.5 | 2.64 (m); 2.52 (m) |
| **13** | 124.6 | 5.32 (m) | 124.6 | 5.32 (m) |
| **14** | 134.5 |  | 134.4 |  |
| **15** | 27.4 | 2.21 (m); 1.97 (m) | 27.3 | 2.21 (m); 1.97 (m) |
| **16** | 27.9 | 1.5 (o); 1.4 (o) | 27.8 | 1.50 (m); 1.39 (m) |
| **17** | 28.6 | 1.5 (o) | 28.6 | 1.47 (m) |
| **18** | 28.9 | 1.6 (o); 1.4 (o) | 28.9 | 1.56 (m); 1.40 (m) |
| **19** | 29.2 | 1.6 (o); 1.4 (o) | 29.2 | 1.60 (m); 1.39 (m) |
| **20** | 34.0 | 1.6 (o); 1.10 (o) | 34 | 1.57 (m); 1.10 (m) |
| **21** | 62.6 |  | 62.6 | 3.29 (m) |
| **22** | 61.9 | 5.02 (m) | 61.7 | 5.00 (m) |
| **23** | 133.2 | 5.58 (m) | 133.2 | 5.58 (m) |
| **24** | 127.6 | 6.55 (m) | 127.6 | 6.55 (m) |
| **25** | 124.8 | 6.5 (m) | 124.8 | 6.50 (m) |
| **26** | 137.5 | 5.65 (m) | 137.3 | 5.65 (m) |
| **27** | 26.6 | 2.53 (m); 2.05 (m) | 26.5 | 2.53 (m); 2.05 (m) |
| **28** | 28.6 | 1.5 (o) | 28.5 | 1.47 (m) |
| **29** | 28.7 | 1.5 (o) | 28.8 | 1.55 (m) |
| **30** | 26.8 | 1.3 (o) | 26.8 | 1.29 (m) |
| **31** | 25.9 | 1.5 (o); 1.4 (m) | 25.9 | 1.58 (m); 1.33 (m) |
| **32** | 33.0 | 1.4 (o) | 32.9 | 1.44 (m); 1.37 (m) |

* Torres et al., *J. Nat. Prod.* **2000**, *63*, 1098-1105. <https://doi.org/10.1021/np9905618>

o: overlapped signals, t: triplet.

# Table S.5. ^1^H (500 MHz) and ^13^C (125 MHz) NMR data of arenosclerin C (2) in CDCl_3_.

| **Arenosclerin C (2) CDCl_3_** | | |
| --- | --- | --- |
| **Position** | **δ ^13^C** | **δ ^1^H** |
| **1** | 51.7 | 3.24 (m); 2.96 (m) |
| **2** | 46.7 | 2.07 (m) |
| **3** | 38.4 | 1.91 (m) |
| **4** | 31.5 | 2.17 (m); 2.03 (m) |
| **5** | 48.1 | 3.45 (m); 3.26 (m) |
| **6** | 58.9 | 2.37 (m); 2.81 (m) |
| **7** | 37.9 | 1.89 (m) |
| **8** | 35.1 | 2.33 (m); 1.21 (m) |
| **9** | 42.4 | 2.14 (m) |
| **10** | 58.3 | 3.59 (m) |
| **11** | 54.9 | 3.21 (m) |
| **12** | 22.0 | 2.64 (m); 2.49 (m) |
| **13** | 122.7 | 5.23 (dt, 11.5, 6.0) |
| **14** | 135.1 |  |
| **15** | 26.4 | 2.21 (m); 1.97 (m) |
| **16** | 27.2 | 1.52 (m); 1.39 (m) |
| **17** | 28.9 | 1.40 (m) |
| **18** | 28.9 | 1.56 (m); 1.41 (m) |
| **19** | 31.5 | 1.60 (m); 1.39 (m) |
| **20** | 35.1 | 1.58 (m); 1.10 (m) |
| **21** | 62.0 | 3.32 (m) |
| **22** | 62.3 | 5.01 (brt, 6.6) |
| **23** | 133.8 | 5.56 (dq, 13.3, 7.4) |
| **24** | 126.9 | 6.42 (q, 10.6) |
| **25** | 123.0 | 6.19 (td, 11.3, 4.2) |
| **26** | 121.8 | 5.67 (m) |
| **27** | 25.8 | 2.46 (m); 2.05 (m) |
| **28** | 29.0 | 1.40 (m) |
| **29** | 27.9 | 1.54 (m) |
| **30** | 26.6 | 1.30 (m) |
| **31** | 25.6 | 1.30 (m) |
| **32** | 32.9 | 1.43 (m); 1.38 (m) |

o: overlapped signals

# Table S.6. [M + H]^+^ ion adducts detected in UHPLC-HRMS analyses of E18-M1 marine sponge extract.

| **Extract** | **UHPLC Rt in min** | ***m/z* of [M + H]^+^ ion adducts (relative abundance in the HRMS)** | **Tentative Identification** |
| --- | --- | --- | --- |
| **E18-M1** | 6.58 | 136.0756 (100 %) | - |
|  | 8.07 | 227.1538 (100 %) | - |
|  | 10.21 | 271.1877 (100 %) | - |
|  | 10.43 | 314.0286 (100 %) | - |
|  | 10.71 | 214.2528 (100 %) | Medelamine A (**3**)  (*m/z* 214.2529) isolated from S*treptomyces* sp.  (Morino et al., 1995). |
|  | 10.81 | 228.2684 (100 %) | Medelamine B (**4**)  (*m/z* 228.2685) isolated from *Streptomyces* sp.  (Morino et al., 1995) |
|  | 11.03 | 262.2297 (100 %). | - |
|  | 11.87 | 256.2997 (100 %) | - |
|  | 12.08 | 290.2608 (100 %) | - |
|  | 12.64 | 301.2161 (100 %) | - |
|  | 13.30 | 303.2317 (100 %) | - |
|  | 14.74 | 341.2660 (100 %) | - |
|  | 15.53 | 282.2789 (100 %) | - |
|  | 16.03 | 609.2915 (100 %) | - |
|  | 16.30 | 535.2704 (100%) | - |
|  | 16.70 | 607.2914 (100 %) | - |


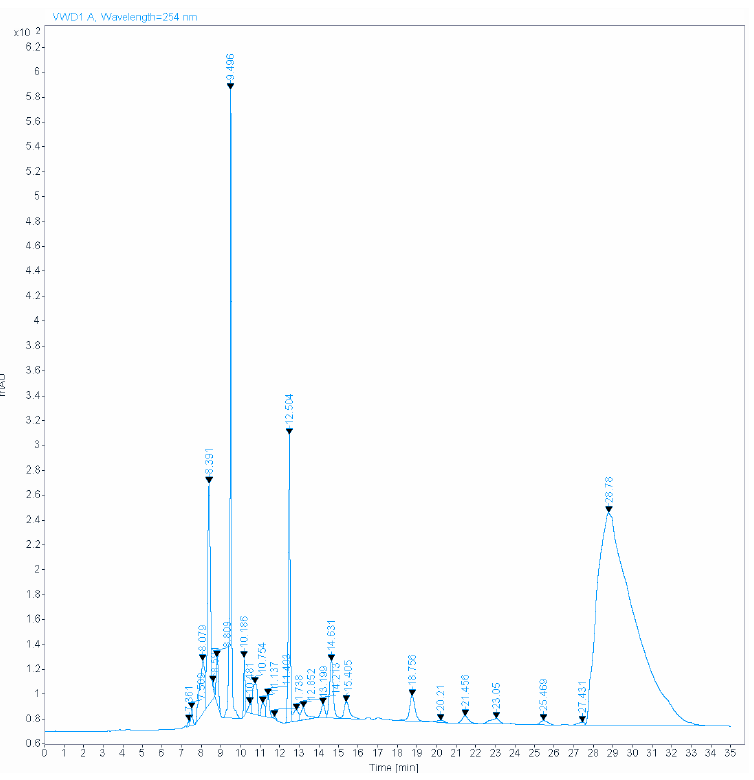


**FM-2-H2**

# Figure S.1. HPLC Chromatogram of subfraction FM-2 from *Haliclona (Rhizoniera) curacaoensis* extract (EY18-4), showing the HPLC peak FM-2-H2 eluted at 9.5 min, where compound 1 was isolated.


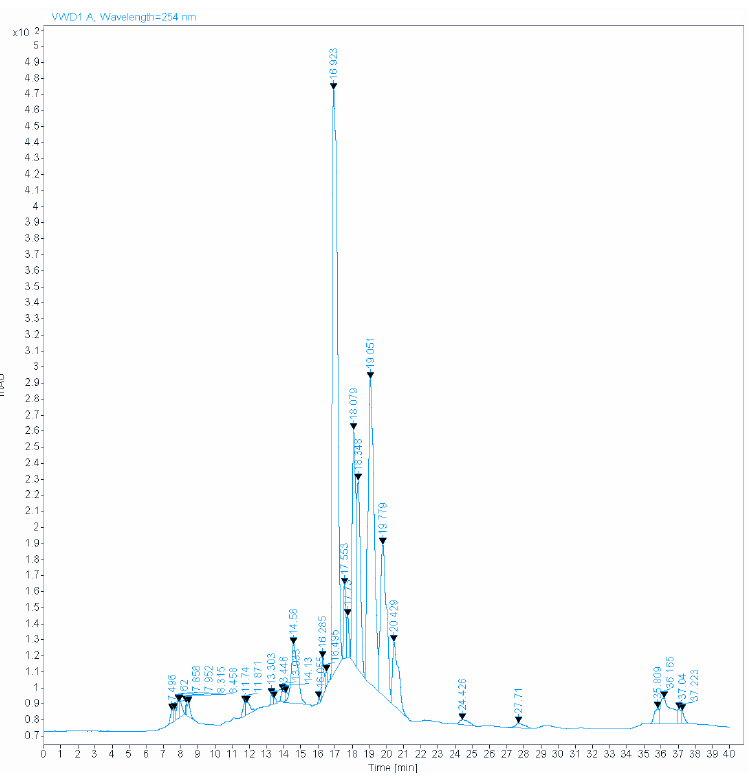


**FD-3-H1**

# Figure S.2. Chromatogram of subfraction FD-3 from *Haliclona* (*Rhizoniera*) *curacaoensis* extract (EY18-4), showing the HPLC peak FD-3-H1 eluted at 16.7 min, where compound 2 was isolated.


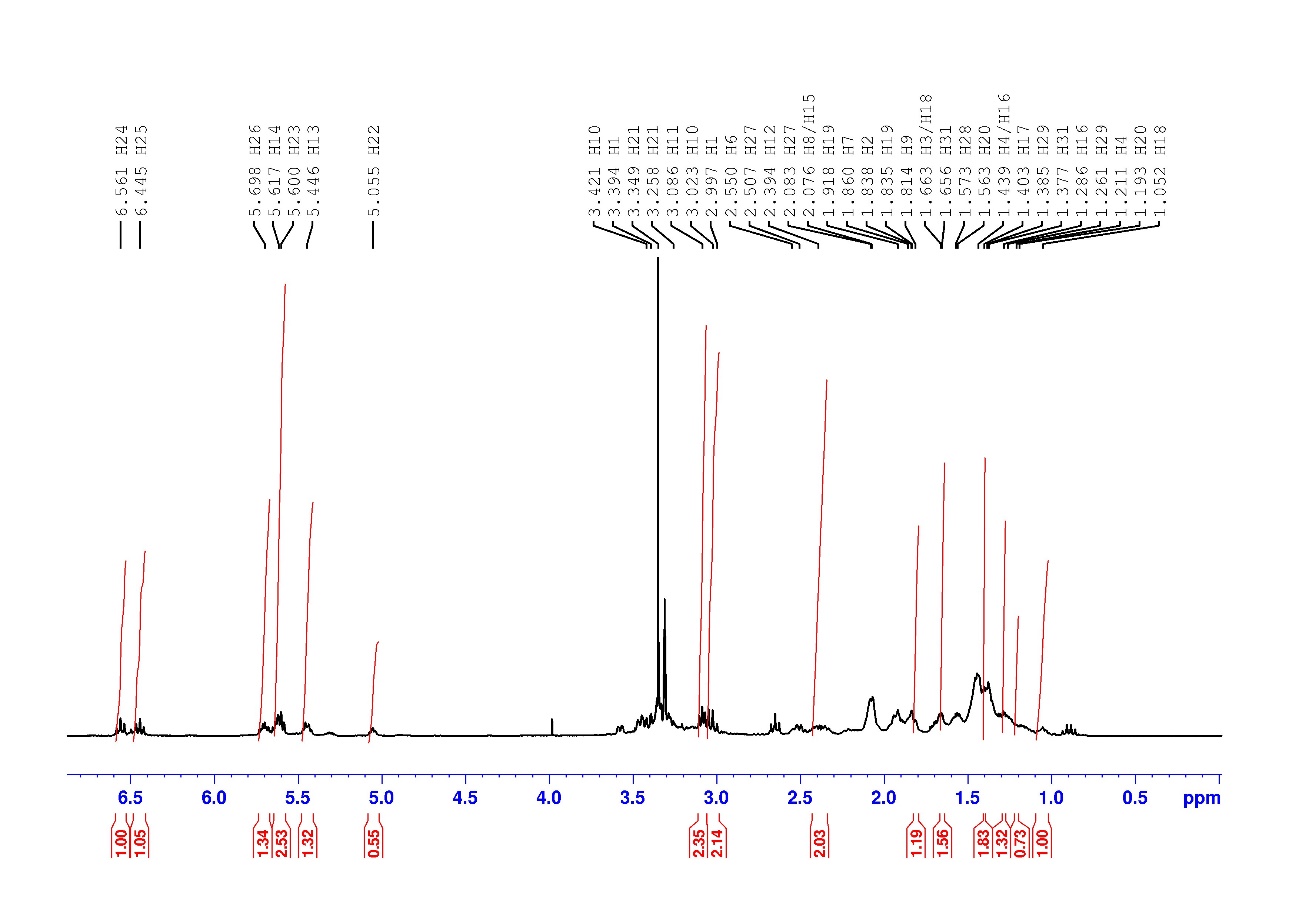


# Figure S.3. ^1^H NMR spectrum (500 MHz, CD_3_OD) of compound 1 (arenosclerin A).


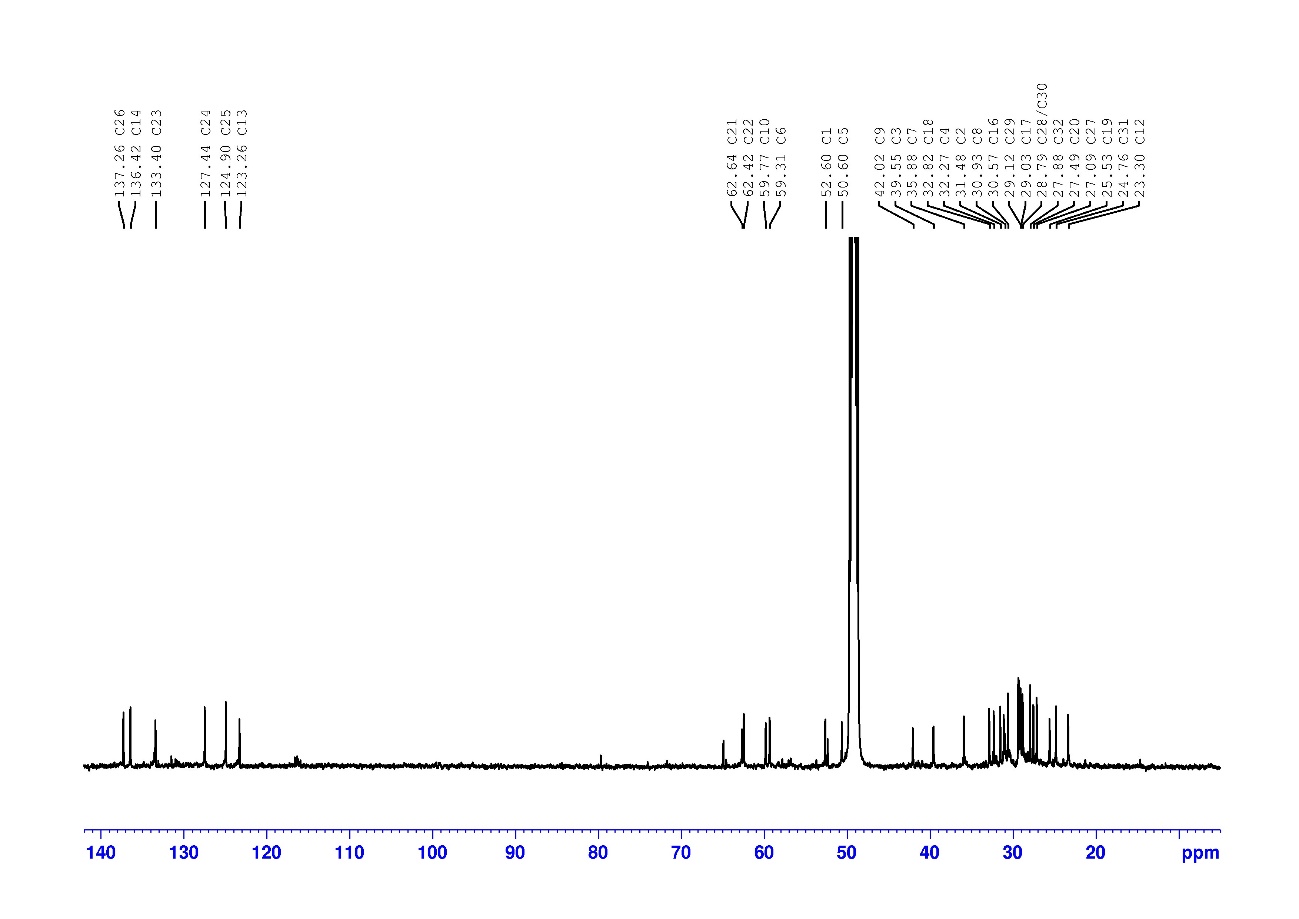


# Figure S.4. ^13^C NMR spectrum (125 MHz, CD_3_OD) of compound 1 (arenosclerin A).


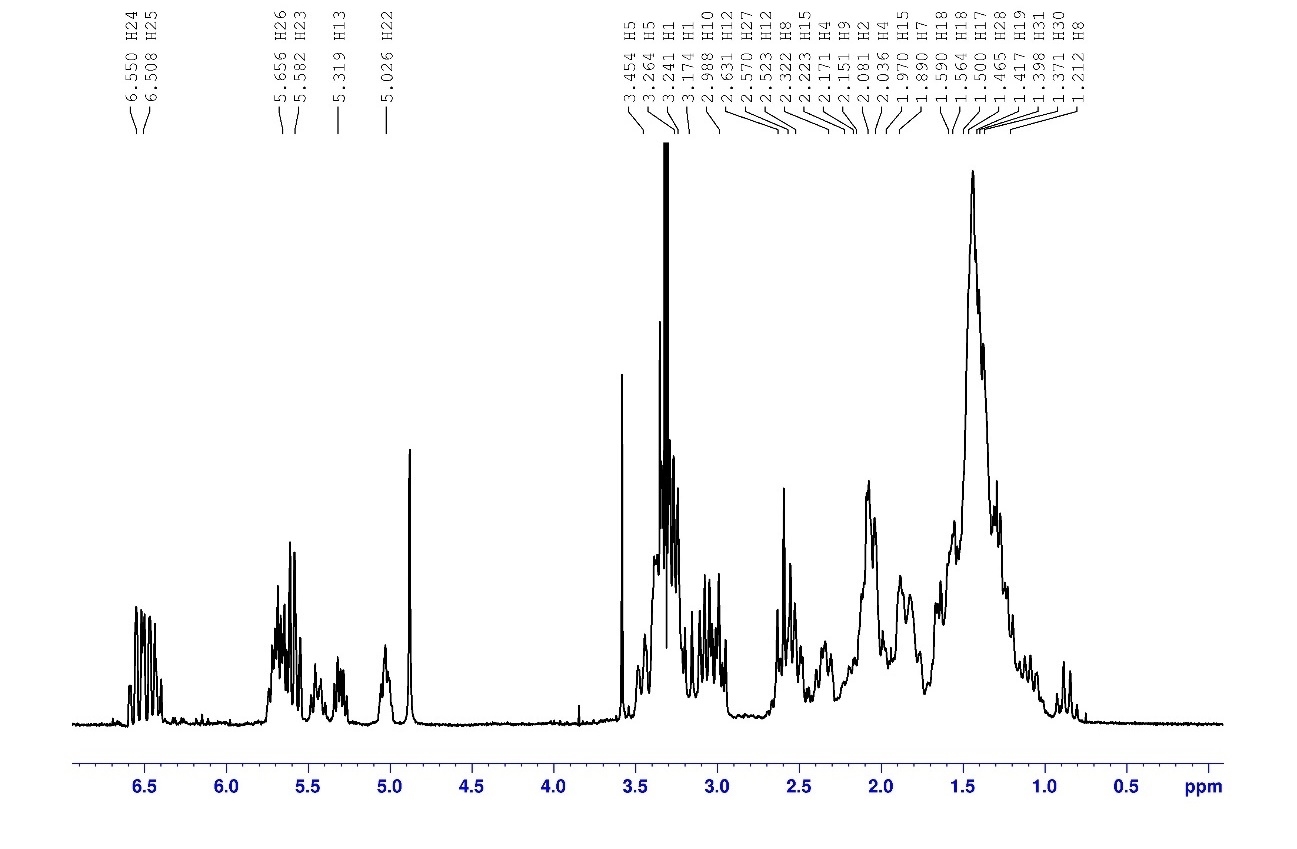


# Figure S.5. ^1^H NMR spectrum (300 MHz, CD_3_OD) of compound 2 (arenosclerin C).


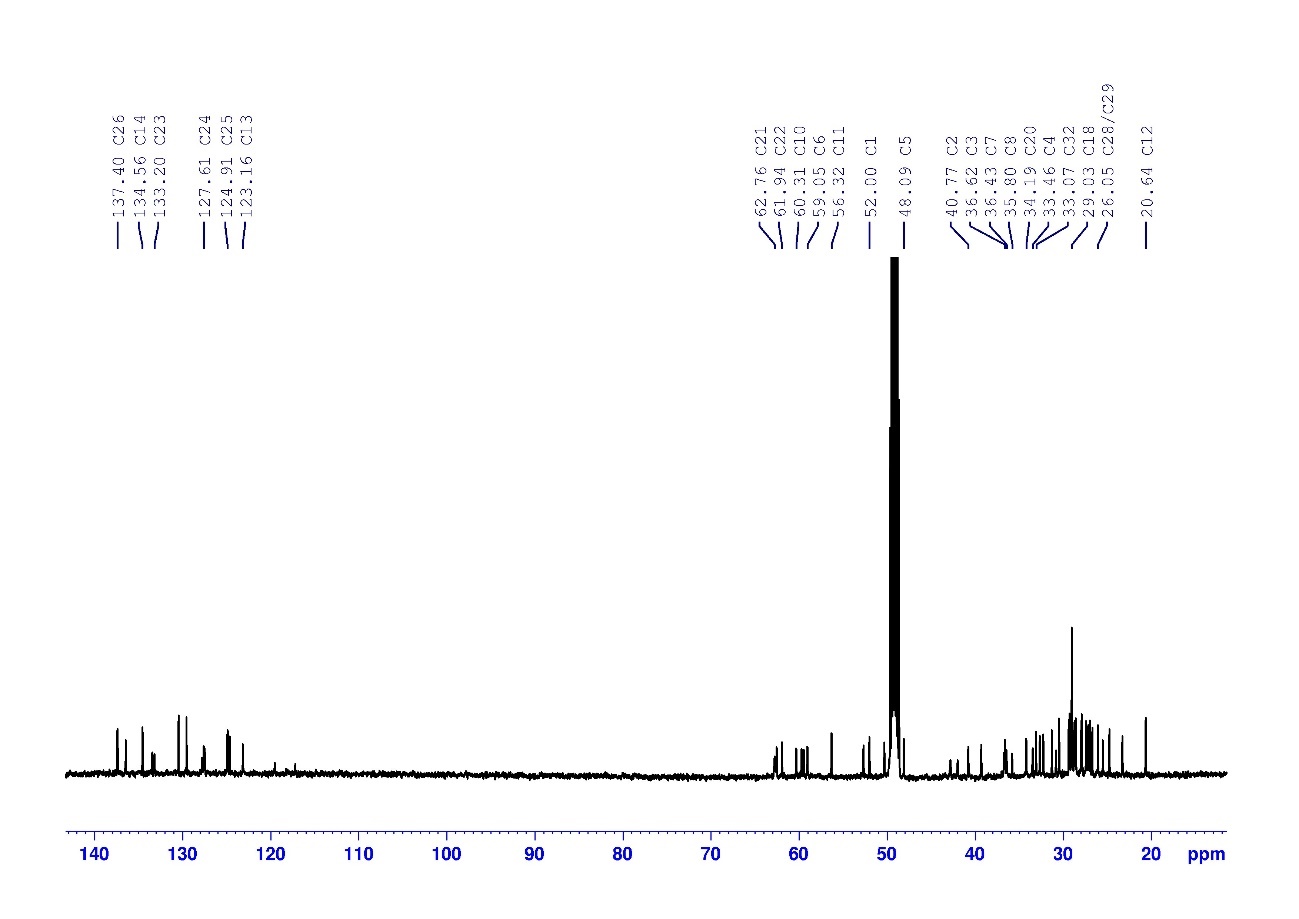


Figure S.6. ^13^C NMR spectrum (125 MHz, CD_3_OD) of compound 2 (arenosclerin C).

*
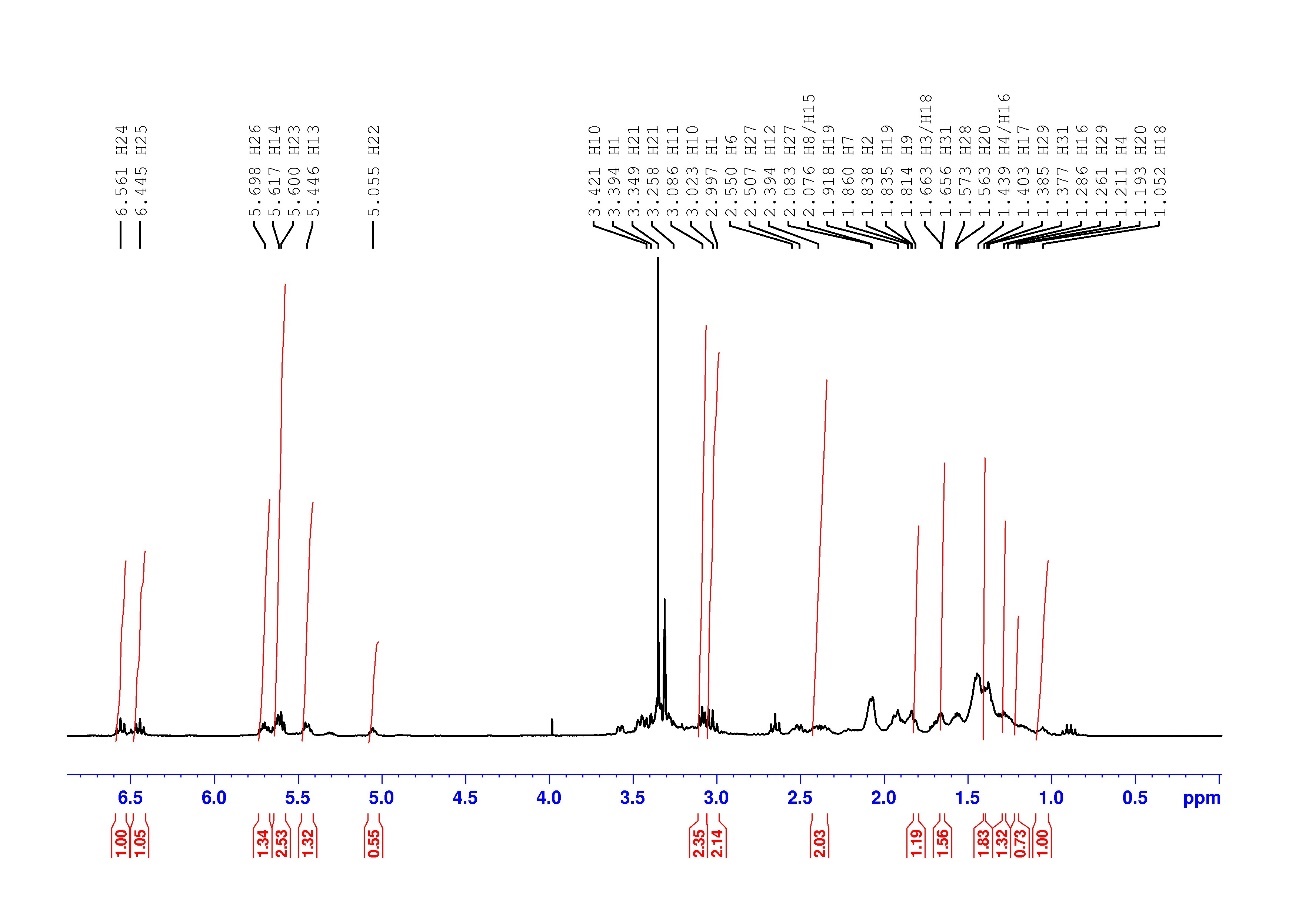
*

**Figure S.7.** ^1^H NMR spectrum (500 MHz, CDCl_3_) of arenosclerin C (**2**).

*
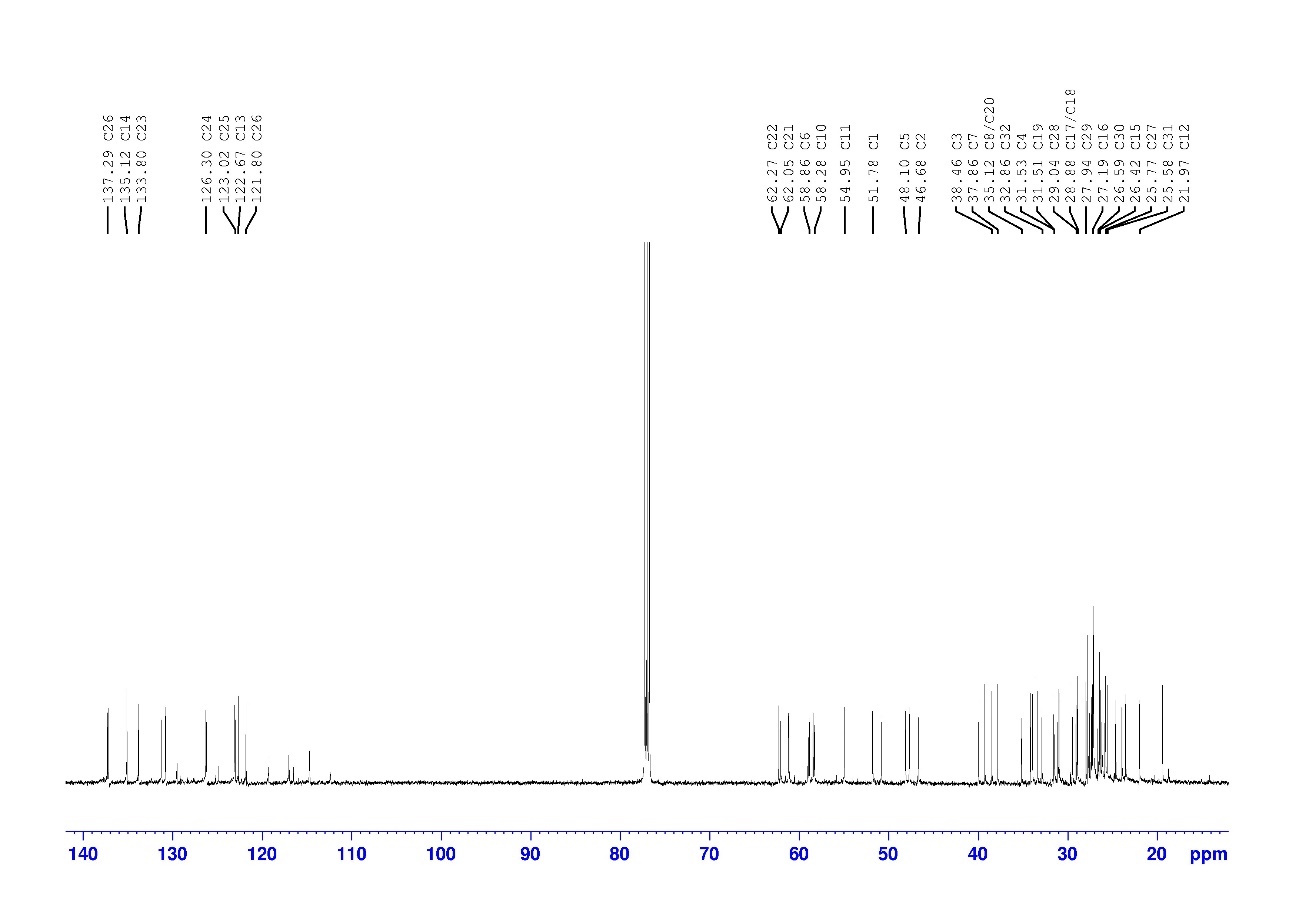
*

**Figure S.8.** ^13^C NMR spectrum (125 MHz, CDCl_3_) of arenosclerin C (**2**).


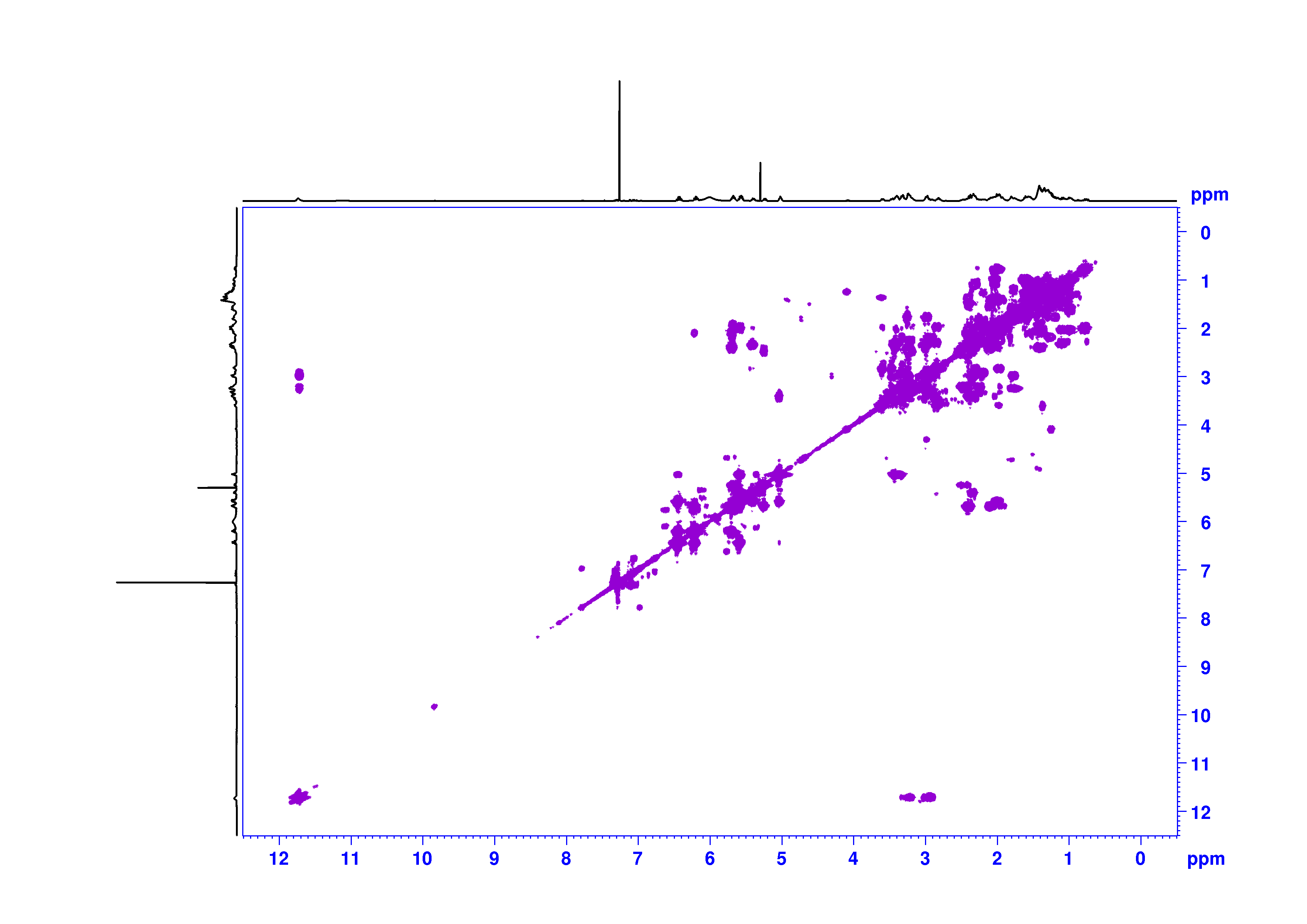


# Figure S.9. ^1^H-^1^H COSY spectrum (500 MHz, CDCl_3_) of compound 2 (arenosclerin C).


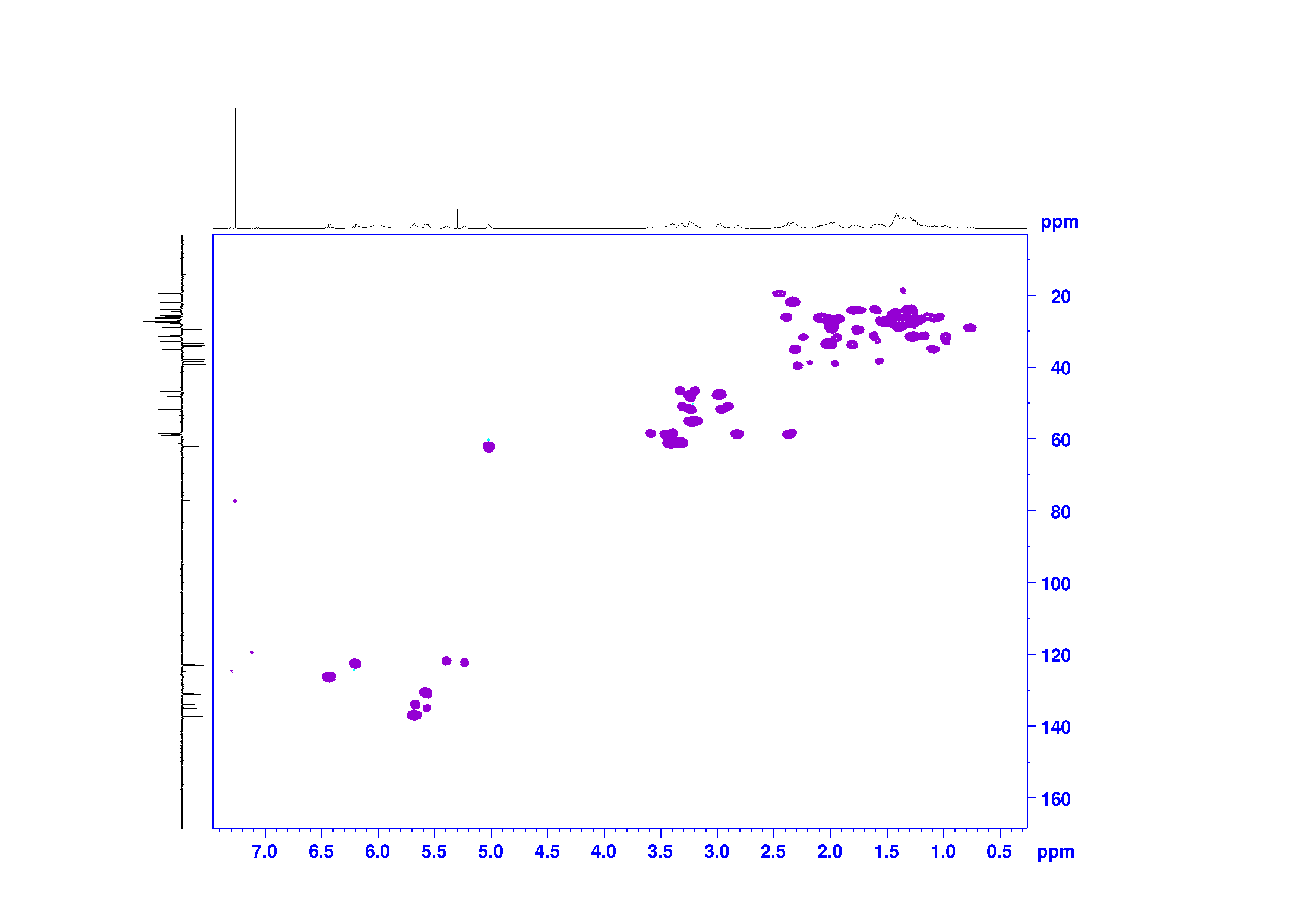


# Figure S.10. HSQC spectrum (500 MHz, CDCl_3_) of compound 2 (arenosclerin C).

| **A** |  |
| --- | --- |
|  | **** |
| **B** |  |
|  | **** |
| **C** |  |
|  | **** |

Figure S.11. UHPLC-HRMS analysis of *Halichondria melanadocia* extract (E18-M1). **(A)** UHPLC chromatogram. HPLC peaks analyzed by HRMS are marked with a red circle. **(B)** HRMS of the HPLC peak eluted at a retention time of 10.71 min, showing a [M + H]^+^ ion adduct corresponding to the molecular formula of C_14_H_31_N, which matched to that of medelamine A (**3**). **(C)** HRMS of the HPLC peak eluted at a retention time of 10.81 min, displaying a [M + H]^+^ ion adduct corresponding to the molecular formula of C_15_H_33_N, which matched to that of medelamine B (**4**).
